# Historical climate change and vicariance events contributed to the intercontinental disjunct distribution pattern of ash species (*Fraxinus*, Oleaceae)

**DOI:** 10.1038/s42003-024-06296-1

**Published:** 2024-05-20

**Authors:** Enze Li, Yushuang Wang, Kangjia Liu, Yanlei Liu, Chao Xu, Wenpan Dong, Zhixiang Zhang

**Affiliations:** 1 Laboratory of Systematic Evolution and Biogeography of Woody Plants, School of Ecology and Nature Conservation, Beijing Forestry University, Beijing, 100083 China; 2 School of Landscape and Ecological Engineering, Hebei University of Engineering, Handan, 056038 China; 3 State Key Laboratory of Systematic and Evolutionary Botany, Institute of Botany, Chinese Academy of Sciences, Beijing, 100093 China

**Keywords:** Plant evolution, Biogeography

## Abstract

The Northern Hemisphere temperate forests exhibit a disjunct distributional pattern in Europe, North America, and East Asia. Here, to reveal the promoter of intercontinental disjunct distribution, *Fraxinus* was used as a model organism to integrate abundant fossil evidence with high-resolution phylogenies in a phytogeographic analysis. We constructed a robust phylogenetic tree using genomic data, reconstructed the geographic ancestral areas, and evaluated the effect of incorporating fossil information on the reconstructed biogeographic history. The phylogenetic relationships of *Fraxinus* were highly resolved and divided into seven clades. *Fraxinus* originated in western North America during Eocene, and six intercontinental dispersal events and five intercontinental vicariance events were occured. Results suggest that climate change and vicariance contributed to the intercontinental disjunct distribution pattern of *Fraxinus*. Moreover, results highlight the necessity of integrating phylogenetic relationship and fossil to improve the reliability of inferred biogeographic events and our understanding of the processes underlying disjunct distributions.

## Abstract (web summary)

A phylogenetic genomics study combining fossil data reveals that climate change led to the intercontinental disjunct distribution pattern of *Fraxinus*

## Introduction

The global distribution of species diversity has been a source of intrigue for biologists since the early 19th century. The underlying mechanisms of these different species distribution patterns are among the most-debated issues in ecology and biogeography^1–4^. Among them, the disjunct distribution pattern is not only one of the most important patterns observed for plants^5–7^, but also for many fungi, arachnids, millipedes, insects, and freshwater fishes^8–11^. Discontinuous distribution implied changes in biogeographic history, on the one hand, investigation on the formation of disjunct distribution patterns helps to understand the drivers of changes in species distribution. On the other hand, exploring disjunct distributed descendants from one ancestor could also shed light on the physiology of species formation^12,13^. Two conditions lead to disjunct distributions. The first is recent dispersal across preexisting geographic barriers, such as long-distance dispersal (LDD)^14^, and the second is vicariance events, such as fragmentation of an continuously ancestral distribution via the formation of new geographic barriers^15^. For example, species of genus *Magnolia* were disjunct distributed in America and Asia^16^.

The main difference between LDD and vicariance events is the formation time of the geographic barriers between regions. LDD refers to the phenomenon where species dispersal cross existing barriers and form the disjunct distribution patterns, which means that the disjunct distribution is formed after geographic barriers^14^. Vicariance events refer to the process of geographic isolation due to geo-climatic events, which leads to the habitat fragmentation of ancestral continuously distributed species, which means that the disjunct distribution is formed no later than geographic barriers^15^. Therefore, the identification of geographic barriers is essential for exploring the formation of disjunct distribution patterns. As for Intercontinental disjunctions patterns, geographic barriers refer to the break-up of land bridges resulting in disconnected relict populations by geographic vicariance, which subsequently have independent evolutionary trajectories on the disjunct continents. For example, the North Atlantic Land Bridge is the “Greenland-Faroes Bridge” that connected Europe and North America^17^ and the Bering Land Bridge connected North America and East Asia^18,19^, resulting in disconnected relict populations by geographical vicariance that have independent evolutionary trajectories on the disjunct continents. On the other hand, historical climate change is also essential factor underlying species intercontinental disjunctions. For example, the vegetation type of the Northern Hemisphere changed from boreo-tropical forest to mixed-mesophotic forest due to drying climates in the Tertiary^20,21^.

Over 100 angiosperm genera exhibit a disjunct distributional pattern in two or more of the following areas: Europe, eastern North America, western North America, and East Asia, with the most notable patterns relating to the connection between East Asia and Eastern North America^22^. However, closely related intercontinental disjunct distribution species exhibit differences in species richness in different regions with similar environmental conditions: Many researchers have compared the species richness of plants between East Asia and eastern North America and found that Asia possesses both higher species richness and higher phylogenetic diversity for angiosperm genera than does North America^23–26^, which also indicates that closely related species from disjunct regions possess different evolutionary, and the formation of disjunct distribution patterns could have played an important role in this process^19^ However, it is unclear how the intercontinental disjunct distribution of the Northern Hemisphere arose. With advances in phylogenomic and biogeographic inference methods, it is possible to integrate historical climate change and evolutionary history to elucidate the formation of Northern Hemisphere intercontinental disjunct distributions.

The genus *Fraxinus* L. (ash) comprises nearly 50 species, which are widely distributed in temperate and subtropical regions of the Northern Hemisphere^27,28^. *Fraxinus* originated in the Eocene (the minimum age of its oldest fossil—a samara of *F. eoemarginata* from the early Eocene in British Columbia, Canada—is around 51.1 Ma^29^)when temperate forests expanded into the Arctic region with global warming. Therefore, the ash lineage was involved in the historical dynamics of the mainly climate-change-driven establishment of Cenozoic forests^29,30^. Extant *Fraxinus* exhibit a disjunct distribution around the Northern Hemisphere, with North America and China being the two main centers of distribution. Recent biogeographic studies have shown that there were multiple intercontinental dispersal events in *Fraxinus*, leading to geographic isolation as well as the formation of new species and lineages^28,31^.

Ancestral area reconstruction requites the basis of species-level phylogenetic researches, robust phylogeny at species level is essential to explore the dynamic history of intercontinental disjunct distribution patterns^32^. However, as woody plants, longer life cycles results in slower evolutionary rates, which also makes the phylogenetic relationships in *Fraxinus* more likely been affected by evolutionary events, such as incomplete lineage sorting^33^. The phylogenetic relationships of *Fraxinus* species remain unresolved based on several chloroplast markers and ITS^27,28,34^. Another potentially promising approach to obtaining an accurate dispersal history is to draw on fossils that directly reveal past occurrences. Several studies have shown that fossil taxa reflect a more complex biogeographic history; for example, the fossil record can expand historical distributions regions, and including fossil information can lead to substantial changes in the inferred biogeographic reconstructions^35,36^. Previous studies suggested that *Fraxinus* originated in Eocene North America and dispersal to Europe after middle Miocene^28,31^. However, *Fraxinus* fossils such as *F. sp* had been discovered in Oligocene strata in Czech Republic^37^. These records challenge the dispersal history inferred in previous biogeographic reconstructions, which solely relied on phylogenetic analyses of extant species to explain the biogeography of *Fraxinus*.

To elucidate the mechanisms driving the disjunct distribution of *Fraxinus* species, it is essential to generate a high-resolution phylogenetic tree and include fossil information. In this study, we aimed to reconstruct the dynamic history of *Fraxinus* distribution patterns. We used chloroplast genome sequences and a nuclear SNP dataset to generate a robust phylogenetic tree. Furthermore, we reconstructed the geographic ancestral areas and evaluated the effect of incorporating fossil information on the reconstructed biogeographic history. In addition to these analyses, we described the dynamic processes from the origins to the current disjunct distribution patterns of *Fraxinus* as related to paleoclimate data.

## Results

### Phylogenomics of *Fraxinus*

**Fig. 1:**
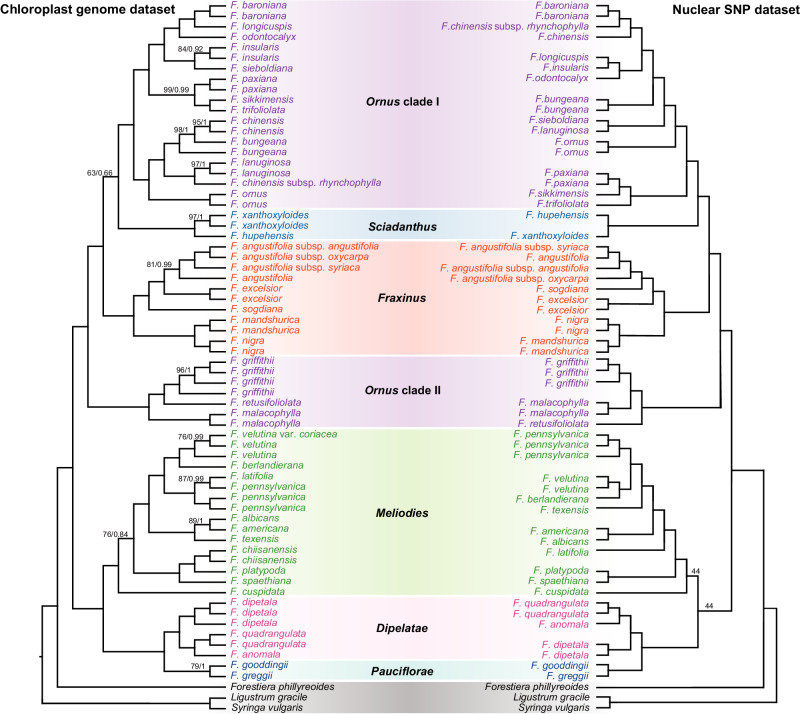
Phylogeny of *Fraxinus* inferred using the chloroplast genome dataset and nuclear SNPs dataset. Numbers associated with nodes indicate ML bootstrap support (BS) values and BI posterior probabilities (PP) in the chloroplast genome dataset or ML bootstrap support (BS) values in the nuclear SNP dataset. BS = 100 and PP = 1 were not shown for simplicity. The different colors represent the six sections of *Fraxinus* according to the traditional classification.

The phylogenetic relationships of *Fraxinus* inferred from the chloroplast genome and nuclear SNP datasets were highly resolved (bootstrap values, BP > 95% and local posterior probability, PP > 0.95 for most nodes in the chloroplast tree, and BP > 95% for most nodes in the nuclear SNP tree) and topologically consistent at the section level. Our study suggested that all *Fraxinus* formed a monophyletic group, with two main lineages (Fig. 1, Supplementary Figs. 1, 2). The first lineage included section *Ornus*, section *Sciadanthus*, and section *Fraxinus*, and section *Ornus* did not form a monophyletic group, having two clades (section *Ornus* I and *Ornus* II). *Ornus* clade I was sister to section *Sciadanthus*, while *Ornus* clade II formed a clade by itself. The second lineage included the section *Melioides*, section *Dipetalae*, and section *Pauciflorae*.

Species trees based on the 5 kb, 10 kb, and 15 kb SNP datasets (Supplementary Figs. 3–5) were congruent with the SNP gene tree. Moreover, there were some incongruences in topology between the chloroplast and nuclear trees at the species level (Fig. 1). Chloroplast–nuclear discordance (or cytonuclear discordance) in plants has generally been attributed to chloroplast capture. Recently, the chloroplast–nuclear discordance in closely related species has been largely explained by organellar introgression, branch length, geography, and incomplete lineage sorting^38–41^. Overall, most nodes of the *Fraxinus* phylogenies based on either the whole chloroplast genome or the nuclear SNPs were highly resolved at the species level, which was important for subsequent estimation of divergence time and inference of biogeographic histories, especially the number of dispersal/vicariance events.

### Divergence time estimation

**Fig. 2:**
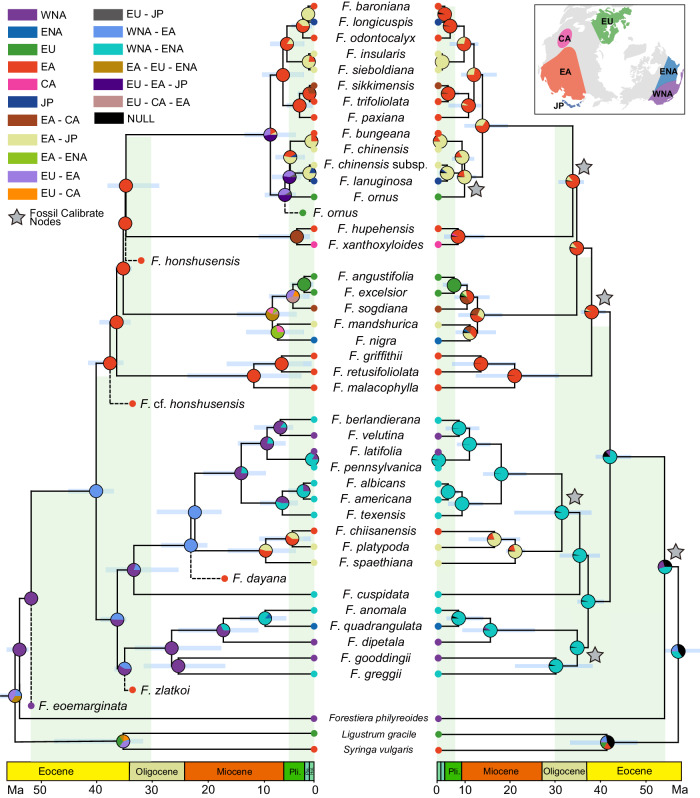
Geographic range evolution of *Fraxinus.* Ancestral distributions at each node of the tip-dating phylogeny of *Fraxinus* generated under a DEC model are on the left, and node-dating results from BAYAREALIKE + J are on the right. Fossil species are marked by dashed lines in the tree, and calibration nodes of the node-dating tree are labeled with gray stars. 95% confidence intervals of time estimations are marked with blue bars at each node. Ancestral regions at different nodes are represented by different colors, and the ranges of the regions are marked in the right corner of the map. Note: WNA western North America, ENA eastern North America, EU Europe, EA East Asia, CA Central Asia, JP Japan. “*F. chinensis* subsp.” refers to *F. chinensis* subsp. *rhynchophylla*.

The results of the node-dating and tip-dating exhibited a high degree of consistency, as exemplified by the tip-dating results: the stem node of *Fraxinus* was dated to 52.78 Ma (95% highest posterior density, HPD: 52.02–53.67 Ma) in the early Eocene, and the two main lineages (the crown node of *Fraxinus*) diverged ~42.33 Ma (95% HPD: 38.13–46.89 Ma) (Fig. 2) in the middle Oligocene. The six sections were estimated to have diverged at 37.04 Ma to 33.25 Ma in the late Eocene and early Oligocene. The time of diversification (the date of the node for the last common ancestor of species) of most species was shown to have been during the middle Oligocene and middle Pliocene (about 28.98–0.18 Ma) (Fig. 2).

### Biogeographic reconstruction without fossil taxa

**Fig. 3:**
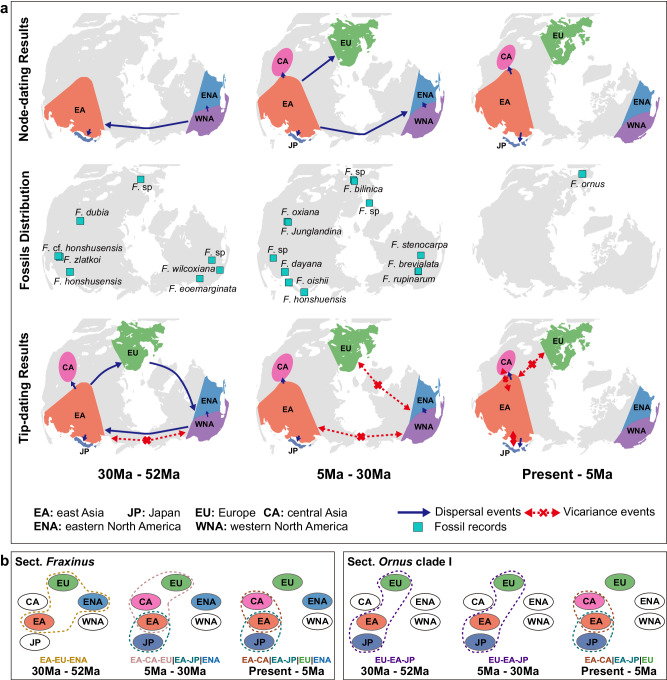
Biogeographic history of *Fraxinus.* **a** Comparison of biogeographic history reconstructed on a node-dating tree and tip-dating tree with fossil distribution history. The unidirectional arrows represent the direction of dispersal events, and the bidirectional arrows represent the vicariance events between regions. **b** Biogeographic histories of section *Fraxinus* and section *Ornus* clade I; both processes were divided into the same three stages as in (**a**). Color-coded areas indicate that species of the section were distributed in this area during the period, multiple regions circled by dashed lines denote the presence of species/ancestor nodes distributed simultaneously in these regions during the period. The text below the image represents the distribution pattern of the section during each period. Note: WNA western North America, ENA eastern North America, EU Europe, EA East Asia, CA Central Asia, JP Japan.

The ancestral area reconstruction based on the node-dating tree supported a North America origin of *Fraxinus* (Fig. 2, Fig. 3a). Biogeographic results indicated that there were five intercontinental dispersal events since the origin of *Fraxinus*. The first dispersal event occurred from North America to East Asia in the middle Eocene, leading to the ancestor of section *Ornus* (clade I and clade II), section *Sciadanthus*, and section *Fraxinus* (Fig. 2, Fig. 3a). The second dispersal event also occurred from North America to East Asia and Japan around the early Oligocene, giving rise to the ancestors of the Asian species of section *Melioides*. All three subsequent dispersal events occurred within 10 Ma to the present—one event from East Asia to eastern North America, and two events from East Asia to Europe (Fig. 2, Fig. 3a).

The node-dating results supported an earlier (Eocene and Oligocene) *Fraxinus* dispersal to East Asia and Japan, whereas *Fraxinus* dispersal to Central Asia and Europe was only supported in recent periods (after the late Miocene). In other words, multiple recent dispersal events resulted in the intercontinental discontinuous distribution pattern of *Fraxinus*. However, these trends were contradictory to the distribution of the fossils (Fig. 3a, Supplementary Table 1). For example, the fossil taxa *Fraxinus* sp. reported from Kundratice, North Bohemia, the Czech Republic^37^ and *Fraxinus dubia* reported from Aktobe, Kazakhstan, in the early Oligocene^42^, support ash dispersal into Europe and Central Asia occurring as early as the early Oligocene. Due to these conflicts, we assumed that the ancestral geographic reconstruction results based on the node-dating tree did not reflect the true picture of the ancestral distribution shifts of *Fraxinus*, and these results were therefore not included in further analyses.

### Biogeographic reconstruction including fossil taxa

Adding fossil taxa radically altered the reconstructed geographic distributions of ash species. The ancestral area reconstruction based on the DEC model^43^ including six fossil occurrences and a tip-dated tree, supported a western North America origin of *Fraxinus* (Fig. 2, Fig. 3a). The biogeographic results indicated 37 dispersal events and seven vicariance events, including six intercontinental dispersal events and five intercontinental vicariance events, in the evolutionary history of *Fraxinus*. According to our results, all six intercontinental dispersal events occurred before 30 Ma. The first intercontinental dispersal event occurred from western North America to East Asia in the middle Eocene, leading to the ancestor of section *Ornus* (clade I and clade II), section *Sciadanthus*, and section *Fraxinus* (Fig. 2). All the other five intercontinental dispersal events occurred rapidly between 35–32 Ma: (i & ii) dispersal events from East Asia via Europe to eastern North America, giving rise to the ancestor of section *Fraxinus*; (iii) a dispersal from western North America to East Asia, giving rise to the fossil taxon *F. zlatkoi*’s ancestors or their related species; (iv) a dispersal from East Asia to Europe, giving rise to the crown ancestors of section *Ornus* clade I; and (v) a dispersal from western North America to East Asia, giving rise to fossil taxon *F*. *dayana*’s ancestors or related species (Fig. 2).

Among the five intercontinental vicariance events, the first vicariance event between East Asia and western North America occurred around 39 Ma, while the last vicariance event between these regions occurred around 21 Ma, which also represented the final break in the dispersal channel between East Asia and North America. The break in the link between Europe and North America occurred around 7 Ma, with one vicariance event. There were two vicariance events between East Asia and Europe, occurring around 5 Ma; these vicariance events occurred mainly in section *Fraxinus* and section *Ornus* clade I (Fig. 3b). According to our results, the ancestors of section *Fraxinus* demonstrated a continuous intercontinental distribution pattern of East Asia-Europe-eastern North America, which was interrupted by the two vicariance events described above, resulting in the intercontinental disjunct distribution pattern. As with section *Ornus* clade I, a single vicariance event led to the disjunct distribution of European species with species in Asian regions (Fig. 3b). Overall, the reconstruction results based on the tip-dating phylogeny supported vicariance events in recent periods leading to the intercontinental disjunct distribution pattern of *Fraxinus*.

### Rhythm of dispersal events consistent with historical climate change

**Fig. 4:**
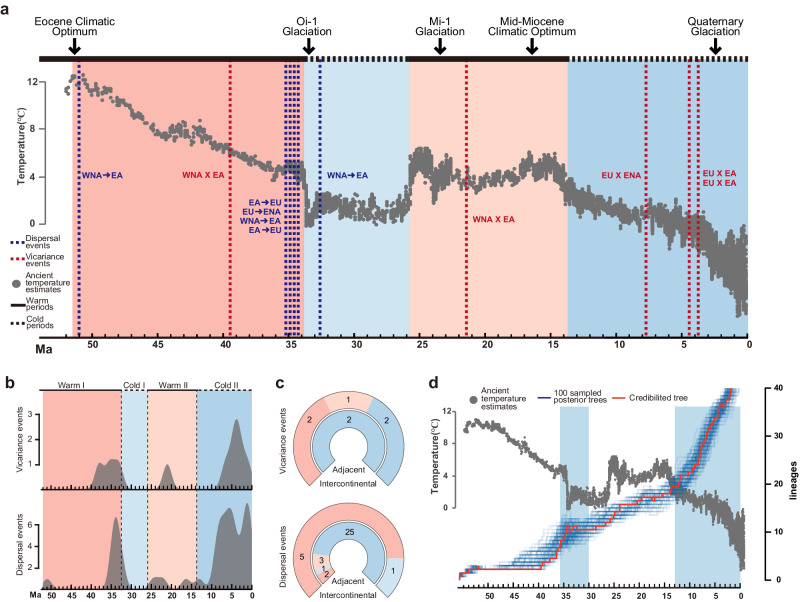
Comparison of *Fraxinus* evolutionary history with the global paleoclimate. **a** Comparison between intercontinental dispersal/vicariance and global paleoclimate. Based on historical temperature changes, we segmented the evolutionary history of *Fraxinus* into “warm periods” (black solid line) and “cold periods” (black dashed line), and the important paleoclimatic periods are denoted at the top of the figure. Historical temperature change results deduced by Zachos are represented by gray dots. Blue dashed lines and red dashed lines denote intercontinental dispersal events and vicariance events, respectively, with specific information marked near the lines (e.g., WNA → EA for dispersal event from WNA to EA, and WNA X EA for vicariance event between those two). **b** Density plots of dispersal events and vicariance events of *Fraxinus*. The entire time scale was divided into four periods: Warm I, Cold I, Warm II, Cold II. **c** Summary of dispersal/vicariance events in four different periods in (**b**). Two intercontinental vicariance events each occurred in Warm I and Warm II. All others occurred in Cold II. There were two dispersal event peaks: one in Warm I, including five intercontinental dispersal events and two adjacent dispersal events, and the other in Cold II, including nine adjacent dispersal events. **d** Comparison of global paleoclimate and lineages through time. Folded lines in blue represent results generated from 100 trees randomly chosen from tip-dating posteriors. Results of the maximum clade credibility tree are shown in the red fold line. Lineages of *Fraxinus* show two rapid rises starting from around 35 Ma and 15 Ma, which were the same periods when the paleoclimate experienced rapid changes.

**Fig. 5:**
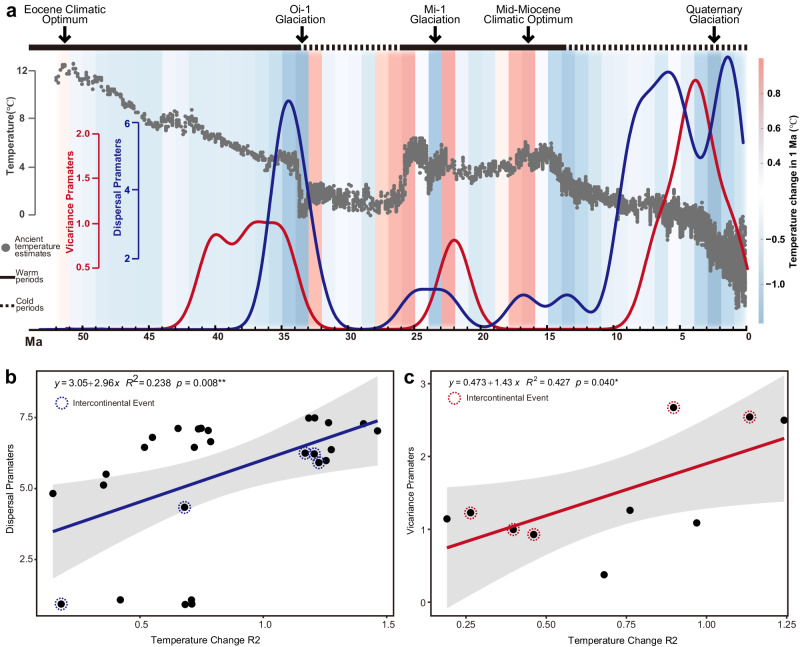
Correlation between climate change and dispersal/vicariance events. **a** Comparison between global temperature change and dispersal/vicariance events. Based on historical temperature changes, we segmented the evolutionary history of *Fraxinus* into “warm periods” (black solid line) and “cold periods” (black dashed line), and the important paleoclimatic periods are denoted at the top of the figure. Historical temperature change results deduced by Zachos are represented by gray dots. Red curve represents the distribution density of vicariance events over time, while the blue curve represents the dispersal events, which were all extracted from RASP. The color changes in the background represent the temperature change per million years. **b**, **c** Results from regression between dispersal/vicariance events and paleoclimate change. Dots representing dispersal density parameters and absolute values of temperature change slopes extracted according to the time of dispersal events. Intercontinental events were labeled with dashed circles, shaded ranges represents 95% confidence intervals. Raw data for this figure could be found in Supplementary Table 10.

The results of comparisons between paleoclimate and evolutionary history are shown in Fig. 4 and Fig. 5. We segmented the evolutionary history of *Fraxinus* into two “warm periods” (black solid line) and two “cold periods” (black dashed line), and important paleoclimatic periods were denoted according to historical temperature changes (Fig. 4a). Combined with the results of the biogeographic history reconstruction, there were two concentrated peaks of dispersal events in *Fraxinus*. One was at the end of warm period I, about 35 Ma, mostly consisting of intercontinental dispersal events (5 in 7), and the other was in cold period II, wholly consisting of adjacent dispersal events (25 in 25). While most vicariance events occurred in cold period II, only two intercontinental vicariance events each occurred in warm period I and warm period II (Fig. 4b, c). At the same time, results from paleoclimate change reconstructions also indicate that periods of dispersal/vicariance events were characterized by relatively large climate shift (Fig. 5a). Regression analyses also indicated that the density of dispersal/vicariance events distributed on the evolutionary history were significantly positively correlated with climate change (Fig. 5b, c). Based on the tip-dating tree, the *Fraxinus* lineage numbers showed two rapid rises starting from 35 Ma and from 15 Ma, which were the same periods in which the paleoclimate experienced rapid changes (Fig. 4d).

## Discussion

Spatiotemporal evolutionary inferences are valuable only if constructed from robust species-phylogenetic relationships and using integrative approaches to elucidating biogeographic history^44^ Compared to previous studies, we inferred robust species-phylogenetic relationships of *Fraxinus* based on whole chloroplast genome sequences and nuclear SNPs, improving the understanding of relationships among ash species. Our findings show that the past distributional history of plant groups has been significantly underrepresented by biogeographic analyses solely based on extant species’ distribution. Although there is a great deal of uncertainty about the phylogenetic position of fossil specimens, this problem was resolved by adding only a small number of fossil specimens. This study emphasizes the vital importance of using genomic data and incorporating fossil data to produce reliable biogeographic analyses.

Including fossils in the biogeographic analysis revealed a western North American origin for *Fraxinus* (Fig. 2). This is supported by paleobotanical evidence^45,46^ The leaves and fruits of *Fraxinus* have been reported from North America in the Eocene, such as *F. wilcoxiana* from the middle-Eocene Claiborne Formation of the southeastern United States and *F. eoemarginata* from the early-Eocene Quilchena locality, Okanagan Highlands, British Columbia, Canada^29,46–48^ while reliable fossil records of this genus in Europe and East Asia prior to the early Oligocene have not been published^45^.

How did *Fraxinus* dispersal occur throughout the northern temperate zone? Our biogeographic reconstruction including fossil species found that *Fraxinus* likely underwent six intercontinental dispersal events and multiple adjacent dispersal events (Fig. 2). In the late Eocene, around 51 Ma—which was close to the Eocene climatic optimum, in which global temperatures were relatively high^49–54^,the ancestor of the section *Ornus* migrated to East Asia. Its migration route probably involved the Bering Land Bridge, in a move possibly facilitated by the similar climates of East Asia and North America^18,19,55^. The fossil records of *F*. *zlatkoi* and *F*. cf. *honshuensis* were reported from the early Oligocene of the Lühe flora, Yunnan Province, Southwest China, providing evidence that the divergence of section *Dipetalae* and section *Ornus* had occurred in Southwest China by the early Oligocene^45^.

The main conflict between our results and the biogeographic scenario described by ref. ^28^ which was based only on analyses of extant species, was the time of *Fraxinus* dispersal to Europe. In the latter study, *Fraxinus* dispersed to Europe during the Miocene, which was similar with our results from node-dating phylogeny, whereas our results from tip-dating phylogeny, which incorporated fossil data, suggest this occurred around 34–35 Ma (Eocene). The latter finding was supported by two fruit fossils from the early Oligocene in the Czech Republic and from the middle Miocene in Iceland^56^, these fossils also supported another dispersal event from Europe to eastern North America (Fig. 3a). Although those fossils were not included in either the tip-dating analysis or the RASP analysis due to their uncertain phylogenetic positions, the fruit fossil in Iceland also supported the likelihood that *Fraxinus* dispersed via the North Atlantic Land Bridge. Our results fully supported the conclusion from Wisniewski et al., which is “Extant species fail to estimate ancestral geographical ranges at older nodes”^36^, fossil records with favorable taxonomic characteristics are essential evidence in historical biogeographical reconstruction. Although there was temporal gap between 50 Ma and 35 Ma in the fossil records covered in this research, new fossils unearthed during this period would only advance the time of *Fraxinus*’s global dispersal and would not affect the history of distribution patterns. In summary, *Fraxinus* likely dispersed throughout the northern temperate zone before the early Oligocene or even before the late Eocene^19,20,57^.

Our results supported the intercontinental disjunct distribution of *Fraxinus* being the result of multiple vicariance events, rather than LDD. Based on the biogeographic history of section *Fraxinus* and section *Ornus* clade I, which demonstrated a history of intercontinental distribution (Fig. 3b), the dispersal channel connecting the entire northern temperate zone did not last: the passage between western North America and Asia was first broken around 21 Ma (Fig. 2), and no further dispersal events occurred between two regions. The dispersal channel between Europe and North America was the second to break, at about 7 Ma, while that linking Europe and East Asia was the last to break, at about 4 Ma (Fig. 3a). After 5 Ma, although there were still adjacent dispersal/vicariance events (Fig. 3a), the main intercontinental range connections had been broken. History of vicariance between intercontinental regions was closely linked to the breakup of Bering Land Bridge and the North Atlantic Land Bridge, which led to the disruption of dispersal corridors. The pattern of disjunct distribution, which was similar to modern patterns, had been established.

Identifying the relative effects of paleoclimatic change on disjunct distributions is an effective approach in macroevolution^58,59^. This study demonstrates the importance of combining paleoclimate and historical vicariance and dispersal events in a phylogenetic approach to discover the dramatic changes in historical climate that drove the dispersal of Northern Hemisphere temperate forests. After comparing the temporal relationship between historical climate change and dispersal/vicariance events, we found a high degree of consistency between the time periods in which dispersal/vicariance events occurred and the time periods in which the climate changed (Fig. 4, Fig. 5).

According to our results, nearly all (5 in 6) intercontinental dispersal events occurred in the warm Eocene, before the Terminal Eocene Event (TEE)^15,52^, which also aligned with the first peak of dispersal events. Although one intercontinental dispersal event occurred about 32 Ma, considering the large temporal gap between the records of Eocene and Oligocene fossils, we presumed this intercontinental dispersal event may have occurred much earlier given more fossil evidence. In the setting of a warmer climate on the time scale, there was one vicariance event between western North America and East Asia after the first dispersal of *Fraxinus*, but the resulting distributional isolation was quickly broken by subsequent dispersal events. The global distribution pattern of *Fraxinus* did not change in the Oligocene except for two dispersal events (Fig. 2, Fig. 3a).

Paleoclimatic changes also supported vicariance events leading to the intercontinental disjunct distribution patterns of *Fraxinus*. In the Miocene, the dispersal channel between East Asia and western North America was broken at about 21 Ma, right after the Miocene Glaciation (Fig. 4a, Fig. 5). Previous hypotheses have suggested that extreme winter temperatures notably limited the northward dispersal of clades^60,61^. In other words, the high-latitude habitat areas that were nearly the only route for *Fraxinus* to disperse along across continents gradually shrank. As the Bering Land Bridge existed at this time period^19^, the main cause of this vicariance event may be due to the temperature drop brought on by the glaciation. After 14 Ma, global temperatures entered a phase of decline, creating low-temperature difficulties for species crossing the high latitude areas^52,54,62,63^. Based on our results, most vicariance events in *Fraxinus* occurred during this period. Although the second peak of dispersal events also occurred during this period, it only consisted of nine adjacent dispersal events and did not include intercontinental dispersal events in high latitude areas (Fig. 4b). The breakage of the link between Europe and North America was the first intercontinental vicariance event of this period. Considering that the North Atlantic Bridge was already very vulnerable by this time due to continental drift, this vicariance event might have been due to both low temperatures and continental drift. With the arrival of Quaternary Glaciation^54,64^, the dispersal channel between East Asia and Europe was broken, and neither intercontinental dispersal nor vicariance events occurred after 5 Ma. Above all, the intercontinental disjunct distribution pattern of *Fraxinus* originated from multiple vicariance events after global distribution. Early warmer climates provided the basis for intercontinental dispersal events of *Fraxinus*, but as temperatures dropped and intercontinental dispersal corridors were weaker or disrupted, the distribution in high latitude regions began to shrink, which promoted the vicariance events and the emergence of intercontinental disjunct distribution pattern.

Our findings show that *Fraxinus* originated in western North America during the Eocene. Based on ancestral area reconstruction, we found a high degree of consistency between the time periods in which dispersal/vicariance events occurred and the time periods in which temperatures were high or low, indicating that global climate change influenced the probability and direction of lineage or species dispersal. The warm climates in the Eocene may have promoted rapid dispersal events of *Fraxinus* via high latitudes to northern temperate continents, while global temperature decline led to the shrinkage of the high-latitude range and closure of dispersal channels between continents, resulting in the intercontinental disjunct distribution pattern of *Fraxinus*. Furthermore, this study supports historical climate change as well as vicariance events impacting the disjunct distribution pattern of temperate deciduous broadleaf forest species such as *Fraxinus*, while acknowledging how the integrative framework of fossil records, robust phylogenetic relationships, and historical climate change information improves our understanding of species distribution history.

## Material & methods

### Sample collection

According to different taxonomic systems, the number of extant *Fraxinus* species ranges from 43 to 60 We assessed the different taxonomic systems, adding the most recently published papers and floras and selected 50 *Fraxinus* species (Supplementary Table 2). We sampled 65 individuals representing 39 species (78% of all species) to reconstruct the phylogeny of *Fraxinus*. 39 samples were collected from the field and from herbarium specimens. The raw sequence data of 21 samples were downloaded from the NCBI SRA database (Supplementary Table 3) to assemble complete chloroplast genomes, while the other five chloroplast genome sequences were downloaded from GenBank. The raw sequence data of three species from other subtribes of Oleaceae were downloaded and used as the outgroup (Supplementary Table 4).

### DNA extraction and sequencing

Total genomic DNA was extracted from silica-dried leaf tissue of living plants and herbarium specimens following a modified CTAB DNA extraction protocol^65^. The DNA from silica-dried tissue was fragmented to construct 350-bp insert libraries, and the DNA from the herbarium material was constructed using 250-bp insert libraries according to the manufacturer’s manual (Illumina Inc., San Diego, CA, USA). Paired-end sequencing was performed on an Illumina HiSeq X-ten at Novogene in Beijing, China.

### Chloroplast genome assembly and annotation

A four-step approach was employed to assemble the chloroplast genome. If following First, adapters were removed and low-quality sequences were trimmed using Trimmomatic version 0.39^66^. Second, remaining high-quality reads were assembled using GetOrganelle v1.7.1^67^. Third, if GetOrganelle was unsuccessful at assembling a complete chloroplast, we used the methods following ref. ^68^. Fourth, Geneious 2021.1.1 was used to check the four junctions between the inverted repeats (IRs) and the small single-copy/large single-copy regions. Complete chloroplast genome sequences were annotated using Plann^69^ and missing or incorrect genes were checked in Sequin. The newly assembled chloroplast genomes were deposited in GenBank (Supplementary Table 5). The whole chloroplast genome was aligned using MAFFT version 7^70^. All alignments were visually inspected with MEGA 11 and manually adjusted as needed^71^.

### Nuclear SNP calling

We modified the pipeline from refs. ^38,72^ to call SNPs from low-depth whole-genome sequencing data. Whole-genome sequences of *Fraxinus excelsior* were used as a ref. ^73^. Finally, whole-genome SNPs were successfully obtained for 71 samples (Supplementary Table 3, Supplementary Table 4). We identified a total of 583,631 high-quality SNPs.

### Phylogenetic analysis

Maximum likelihood (ML) and Bayesian inference (BI) methods were used to infer the phylogenetic relationships of *Fraxinus* using the whole chloroplast genome dataset. The ML tree was generated using RAxML-NG^74^ with the best-fit model determined using ModelFinder^75^, and branch support was assessed with 1000 regular bootstrap replicates. BI analyses of chloroplast genomes were conducted with Mrbayes version 3.2^76^. Two independent Markov Chain Monte Carlo (MCMC) analyses were performed, each of them runs with three heated and one cold chain for 20 million generations, with sampling for every 100^th^ tree, starting with a random tree. The initial 20% of the samples were discarded as burn-in to confirm the stationarity, and when the average standard deviation of split frequencies was <0.01, the result was considered to reach stationarity.

For the SNP dataset, two methods were used to generate phylogenetic trees. The first was the ML method, which was produced using RAxML under the best-fit model (GTR + ASC). The second was the subsampling method, by which the SNP dataset was randomly sampled with replacement to create subsets of 5 kb, 10 kb, and 15 kb sites, each length was repeated 1,000 times, and each small SNP dataset was used to infer a gene tree using RAxML-NG. Species trees were reconstructed by summarizing gene trees using ASTRAL-III^77^ and the quartet scores were calculated in ASTRAL to examine the number of gene tree quartets supporting the primary topology.

### Selection and incorporation of extinct taxa

To evaluate the impact of fossil occurrence on biogeographic inference, we incorporated extinct taxa distribution information by assigning fossils to the phylogenies iteratively, based on their taxonomic placement. The fruits of *Fraxinus* show more structural consistency and are easily recognized, and hence more reliably determined to the generic level in the fossil record^45^. Moreover, in order to accurately place fossil species on the phylogeny, we filtered the dataset for the most reliable fossil records by applying the following criteria: (1) the fossil is a fruit, (2) the fossil has been assigned to a modern section, and (3) the fossil has been described in detail in comparison with modern species. Six fossil taxa and one fossil of an extant species were filtered for subsequent analyses. To verify the robustness of fossil lineages’ location in the phylogeny. We performed principal component analysis (PCA) based on the fruit-seed morphology datasets from ref. ^30^ as well as fossil morphology dataset. Detailed information on the fossils and morphology datasets are presented in Supplementary Table 6 and Supplementary Table 7.

The samara of *F. eoemarginata* Mathewes, S. B. Archibald et A. Lundgren found in early-Eocene sediments^29,46^, which is the oldest reliable *Fraxinus* record, was placed as a sister group to all the extant *Fraxinus* species. The fossil of *F. zlatkoi* Meng-Xiao Wu et J. Huang, found in Lühe, Yunnan, China^30^, which belongs to the section *Dipetalae*, was placed as the stem group of section *Dipetalae*. The fossil of *F*. cf. *honshuensis* Tanai et Onoe^30^ from Lühe, Yunnan, China, was placed as a sister group to the section *Ornus*. The fossil of *F. honshuensis* Tanai et Onoe s^78^ from the Oligocene, and its closest extant relative was reported to be *F. chinensis* subsp. *rhynchophylla* (Hance) A.E.Murray. Based on the phylogenetic relationships in *Fraxinus* (see the results), we placed it as the stem of section *Ornus* clade I. The closest extant relative of the fossil *F. dayana* R. W. Chaney et Axelrod^79^ was reported to be *F. platypoda* Oliv., which belongs to the section *Melioides*. We therefore placed *F. dayana* as a sister group to section *Melioides*. The results from PCA also supported the above classification (Supplementary Fig. 6). The fossil fruit record of *F. ornus* L.^80^ from Bernasso, France, was placed as a stem node of this extant species.

### Time-calibrated phylogeny

To estimate the divergence time of *Fraxinus*, we reduced the sampling number to 54 with each sample for a species. To assess possible calibration incongruence, two methods of dating—node-dating and tip-dating—were used to estimate the divergence times of Oleaceae. For the node-dating, seven node calibration points were used as priors. The other seven priors were the seven fossils of *Fraxinus* (Supplementary Table 5). A crown age of 52.5 Ma for tribe Oleeae was used in accordance with the results of ref. ^51^ calibrated analysis.

The divergence-time estimation was performed using BEAST version 2.6.3^81^. We ran two analyses with uncorrelated lognormal distribution-relaxed molecular clock models to account for rate variability among lineages: the Yule speciation model (node-dating) and the Fossilized Birth–Death Model (tip-dating). Each analysis ran for 500,000,000 generations using the MCMC method, sampling trees every 10,000 generations. Details of prior parameter settings are shown in Supplementary Table 6. The stationary phase was examined through Tracer version 1.7.1^53^ to evaluate convergence and to ensure that the sufficient and effective sample size for all parameters surpassed 200. A burn-in of 10% of generations was discarded, and TreeAnnotator version 2.6.3 was used to produce a maximum clade credibility tree for each analysis method.

### Biogeographic analysis with and without fossil taxa

To fully demonstrate the impact of the fossil record on ancestral area reconstructions, the node-dating tree and tip-dating tree were used for biogeographic reconstruction. To characterize the historical distribution of *Fraxinus*, we obtained additional fossil records from the literature (Supplementary Table 1). We then divided the overall distributional range of *Fraxinus* into six biogeographic regions: ENA (eastern North America), WNA (western North America), JP (Japan), EU (Europe), EA (East Asia) and CA (Central Asia). This division was mainly based on areas of endemism for *Fraxinus* and the existence of water or plateau barriers between continents.

To increase the robustness of the results, we ran model tests for the node-dating phylogeny and tip-dating phylogeny separately to find the best model based on the corrected Akaike information criterion. Previous results have shown that the best model for node-dating trees is BAYAREALIKE + J, while the best model for tip-dating trees is the Dispersal-Extinction-Cladogenesis (DEC) model^43^ (Supplementary Table 8). In order to exclude the effects from different models, we ran analysis of both models on both phylogeny (Supplementary Figs. 7, 8). To reflect the major changes in continental connectivity, different basic dispersal probabilities among regions were set to reflect inter-region connectivity. The dispersal constraints were divided into three versions (>30 Ma, 30–5 Ma, 5–0 Ma) according to the history of continental drift and climate change: Before 30 Ma, the North Atlantic land bridge and the Bering land bridge remained stable, thus we set the dispersal constraints between WNA and EA, ENA, and EU remained 1. During 30Ma-5Ma, North Atlantic land bridge became weaker, we set the dispersal between EU and WNA to 0.5. After 5 Ma, the global climate became substantially colder and more seasonal. North Atlantic land bridge nearly break while the Bering land bridge was weaker, therefore, the dispersal constraints between ENA and EU was set to 0.25, and the dispersal constraints between WNA and EA was set to 0.5^54^ (Supplementary Table 9). All analyses were implemented in RASP (reconstruct ancestral state in phylogenies) version 4.2^32^ using the dated phylogeny and tree containing extant and extinct species. Dispersal/vicariance events were extracted from the BioGeoBEARS module in RASP.

### Correlation analysis of evolutionary history and global paleoclimate change

Most studies suggest that the formation of disjunct distribution patterns between Asia and North America was related to changes in the global paleoclimate^19,22^. To explore the relationship between the paleoclimate history and the evolutionary history of *Fraxinus*, we compared the dispersal/vicariance events with historical temperature variation at the same time scale. A dataset of global deep-sea temperature and its variance was used to represent the historical changes in global temperature^82^. To demonstrate changes in lineage numbers over the evolutionary history of all *Fraxinus*, we ran a Lineage-Through-Time analysis using the “ape” package for R^83^ based on the maximum clade credibility tree, as well as 100 credibility trees randomly chosen from BEAST datasets.

### Statistics and reproducibility

To further explore the relationship between dynamics of paleoclimate change and dispersal history of *Fraxinus*. We fitted a global temperature change curve using loess based on the temperature scatter values provided by ref. ^54^. Then, we employed the different method in R to calculate the slopes of temperature change at the time of each dispersal/vicariance event, taking the absolute value of the slopes as the intensity of climate change during that period. In order to quantify the distribution of dispersal/vicariance events during the whole evolutionary history of *Fraxinus*, we obtained the corresponding event density parameter from the “Diagram” interface in the RASP results^32^. Finally, we conducted ordinary least squares regression to calculate the correlation between climate change and dispersal/vicariance events, with each event treated as a degree of freedom (Supplementary Table 10). All these analyses were performed in R version 4.0.3.

### Supplementary information

Supplementary information

## Data Availability

The newly sequenced raw reads of this study are deposited the GenBank database under the BioProject PRJNA1103399 and PRJNA820313. Details and other downloaded accessions can be found in Supplementary Tables 3 and 5. All supplementary files can be found in Supplementary Information.

## Code availability

The code used for generating the randomized sampled SNP datasets could be accessed through Notion: https://foil-stomach-0a4.notion.site/Random-sampling-34c4efd3336e440693e7126a38ea1216?pvs=4.
