# Supplementary material for: Historical climate change and vicariance events contributed to the intercontinental disjunct distribution pattern of ash species (Fraxinus, Oleaceae)
Source: Commun Biol. 2024 May 20;7:603. doi: 10.1038/s42003-024-06296-1 (PMC11106067; doi:10.1038/s42003-024-06296-1)
Supplement: Supplementary file 1 — Supplementary information [file 42003_2024_6296_MOESM1_ESM.pdf]

Fossils and high-resolution phylogenies revealed the phytogeographic history of ash species (Fraxinus, Oleaceae)

Enze Li[1], Yushuang Wang[1], Kangjia Liu[1], Yanlei Liu[2], Chao Xu[3], Wenpan Dong[1]*, Zhixiang Zhang[1]*

1 Laboratory of Systematic Evolution and Biogeography of Woody Plants, School of Ecology and Nature Conservation, Beijing Forestry University, Beijing 100083, China

2 School of Landscape and Ecological Engineering, Hebei University of Engineering, Handan 056038, China

3 State Key Laboratory of Systematic and Evolutionary Botany, Institute of Botany, Chinese Academy of Sciences, Beijing 100093, China

* Corresponding authors: Wenpan Dong: wpdong@bjfu.edu.cn

Zhixiang Zhang: zxzhang@bjfu.edu.cn

SUPPLEMENTARY INFORMATION

Supplementary Tables and Figures

Supplementary Table 1. Selected fossil records of *Fraxinus* in Figure 3.

	Age	Location	Taxa	Reference
Early Eocene	51.1-51.9 Ma	Quilchena locality, British, Columbia, Canada	Fraxinus eoemarginata	Mathewes et al. 2021
Middle Eocene	-	western Tennessee, USA	F. wilcoxiana	Call and Dilcher 1992
Early Oligocene	31-35 Ma	Luhe, Yunnan, China	F. zlatkoi	Wu et al. 2022
Early Oligocene	31-35 Ma	Luhe, Yunnan, China	Fraxinus cf. honshuensis	Wu et al. 2022
Early Oligocene	31.93-33.57 Ma	Kundratice, North Bohemia, the Czech Republic	Fraxinus sp.	Kvaček and Walther 1998
Early Oligocene	-	Bridge Creek, Oregon, USA	Fraxinus sp.	Meyer and Manchester 1997
Early Oligocene	-	Aktobe, Kazakhstan	Fraxinus dubia	Zhilin et al. 1989
Middle Oligoceae	-	Haynes Creek, Idaho, USA	Fraxinus stenocarpa	Axelrod 1998
Late Oligocene	-	Beaverhead, Montana, USA	Fraxinus brevialata	Becker 1969
Late Oligocene	-	Beaverhead, Montana, USA	Fraxinus rupinarum	Becker 1969
Oligocene	31.44 Ma	Longjing, Jilin, China	Fraxinus honshuensis	Guo and Zhang 2002
Early Miocene	-	Maguan, Yunnan, China	Fraxinus sp.	Jia 2017
Early Miocene	-	Alkare, Kazakhstan	Fraxinus juglandina	Bruch and Zhilin et al. 2007
Early Miocene	-	Yuzhny, Sarekum, Kazakhstan	Fraxinus oxiana	Zhilin et al. 1989
Early Miocene	-	Bohemia, the Czech Republic	Fraxinus bilinica	Kvaček and Teodoridis 2007.
Early Miocene	-	Wurzburg, Austria	Fraxinus sp.	Kvaček et al. 2001
Middle Miocene	15.5-17 Ma	Shanwang, Shandong, China	Fraxinus dayana	Yang and Yang 1994
Middle Miocene	-	Samakesaroma, Hokkaido, Japan	Fraxinus honshuensis	Tanai and Suzuki 1965
Middle Miocene	-	Pohang, South Korea	Fraxinus oishii	Jung and Lee 2009
Middle Miocene	13.5–15 Ma	Tafla, Ketileseyri, Iceland	Fraxinus sp.	Grímsson et al. 2007
Late Pliocene	-	Bernasso, France	Fraxinus ornus	Leroy and Roiron 1996

Supplementary Table 2. Classification of *Fraxinus* (Oleaceae) used in the study and their geographical distributions.

Sections	Species	Synonyms used in this study	Distribution
Dipetalae	anomala Torr. ex S. Wats.		SW USA, N Mexico
Dipetalae	dipetala Hook. et Arn.	trifoliata (Torr.) Lewis & Epling	SW USA, N Mexico (Baja California)
Dipetalae	quadrangulata Michx.		E & C USA, C Canada
Fraxinus	angustifolia Vahl. ssp. angustifolia	monophylla Desf.	SW Europe
Fraxinus	angustifolia Vahl. ssp. oxycarpa oxycarpa Willd., pallisiae A. J.		SE Europe (M.Bieb. ex Willd.) Franco & Rocha Afonso Willmott, obliqua Tausch
Fraxinus	angustifolia Vahl. ssp. syriaca (Boiss.) Yalt.	potamophila Herder, holotricha Koehne, syriaca Boiss.	W & E Asia (Turkey to Pakistan and Russia) and Algeria
Fraxinus	excelsior L.	turkestanica Carrie`re	C & N Europe
Fraxinus	mandshurica Rupr.		E Asia (China, Japan, Korea, E Russia)
Fraxinus	nigra Marsh.		E USA, E Canada
Fraxinus	sogdiana Bunge		Turkey to C Asia
Melioides	albicans Buckl.		SW USA (Oklahoma, Texas), N Mexico
Melioides	americana L.	biltmoreana Beadle	E USA, E Canada
Melioides	berlandieriana A. DC.		SW USA, NE Mexico
Melioides	caroliniana Mill.		SE USA
Melioides	chiisanensis Nakai		Korea
Melioides	cuspidata Torr.		SW USA, Mexico
Melioides	latifolia Benth.		W USA

Melioides	papillosa Lingelsh.		SW USA (SE Arizona, SW New Mexico, Texas), Mexico (W Chihuahua, NE Sonora)
Melioides	pennsylvanica Marsh.	richardii Bosc	C & E USA, Canada
Melioides	platypoda Oliv.		E Asia
Melioides	profunda (Bush) Bush	tomentosa Michx. f.	E USA
Melioides	spaethiana Lingelsh.		Japan
Melioides	texensis A. Gray		S USA (Texas)
Melioides	uhdei (Wenzig) Lingelsh.		Guatemala, Honduras, Mexico, USA (Hawaii, Puerto Rico)
Melioides	velutina Torr		SW USA, N Mexico
Ornus	apertisquamifera Hara		Japan
Ornus	baroniana Diels		China
Ornus	bungeana DC		China
Ornus	chinensis Roxb.		China, Japan, Korea, Vietnam
Ornus	floribunda Wall.	retusa Champ. ex Benth.var. henryana	C & E Asia (from Afghanistan to Japan)
Ornus	griffithii G. B. Clarke		E Asia (from NE India to Japan and Indonesia)
Ornus	insularis Hemsl.		China, Japan
Ornus	lanuginosa Koidz. var. lanuginosa and var. serrata (Nakai) Hara		Japan
Ornus	longicuspis Sieb. & Zucc.		Japan
Ornus	malacophylla Hemsl.		China, Thailand
Ornus	micrantha Lingelsh.		C Asia (Punjab to Nepal, Himalayas)
Ornus	odontocalyx Handel-Mazzetti ex E. Peter,		China

Ornus	ornus L.		Mediterranean area, N Africa and SW Asia
Ornus	paxiana Lingelsh.		Himalayas, China
Ornus	raibocarpa Regel		C Asia (Turkestan mountains, Iran, Pakistan, Afghanistan)
Ornus	retusifoliolata Feng ex P. Y. Bai		China, Thailand
Ornus	sieboldiana Blume	mariesii Hook. f.	China, Japan, Korea
Ornus	sikkimensis (Lingelsh.) Handel-Mazzetti		Himalaya, China
Ornus	stylosa Lingelsh.		China
Ornus	trifoliolata W. W. Smith		China
Pauciflorae	dubia		Mexico, Guatemala
Pauciflorae	gooddingii Little		SW USA (Arizona), N Mexico
Pauciflorae	greggii A. Gray		SW USA, Mexico
Pauciflorae	purpusii Brandegee		Mexico, Guatemala
Pauciflorae	rufescens Lingelsh.		Mexico
Sciadanthus	hubeiensis S. Z. Qu, C. B. Shang & P. L. Su	hoopiensis	China
Sciadanthus	xanthoxyloides (G. Don) DC.		N Africa (Algeria, Morocco) to Asia (Afghanistan to China)

Supplementary Table 3. The samples used in this study. Collection locality and voucher information are provided for newly sequenced samples. All samples except those only have assemble on NCBI were used to generate for SNP sequences.

Species	Section	Source of materials	Voucher	Accession number of SRA	Accession number of chloroplast genome	Locality
F. lanuginosa	*Ornus*	This study	BOP132360	SRR28778854		Aomori-shi, Japan
F. dipetala	*Dipetalae*	This study	BOP132348	SRR28778061		Baja California, Mexico
F. excelsior	*Fraxinus*	This study	POC516253	SRR18497188		Beijing Botanical Garden, Beijing, China
F. pennsylvanica	*Melioides*	This study	POC516251	SRR28787792		Beijing Botanical Garden, Beijing, China
F. baroniana	*Ornus*	This study	POC516242	SRR28787795		Beijing Botanical Garden, Beijing, China
F. bungeana	*Ornus*	This study	POC516247	SRR28787793		Beijing Botanical Garden, Beijing, China
F. hupehensis	*Sciadanthus*	This study	POC516254	SRR18497187		Beijing Botanical Garden, Beijing, China
F. velutina var. *coriacea*	*Melioides*	This study	BOP132490	SRR28781563		California, USA
F. chinensis	*Ornus*	This study	POC506018	SRR28787795		Dalian, Liaoning, China
F. texensis	*Melioides*	This study	ENC850139	SRR28802820		Florida, USA
F. platypoda	*Melioides*	This study	BOP132432	SRR28778850		Gansu, China
F. berlandieriana	*Melioides*	This study	BOP132272	SRR28777864		Gomez Palacio, Torreon, Mexico
F. chinensis subsp. *rhynchophylla*	*Ornus*	This study	BOP132451	SRR18497230		Gyeongsangnam-do, Korean
F. insularis	*Ornus*	This study	BOP132443	SRR28778849		Hongkong, China
F. griffithii	*Ornus*	This study	BOP132327	SRR28778062		Ishigaki-jima, Japan
F. insularis	*Ornus*	This study	ENC850137	SRR28802822		Jingan, Jiangxi, China
F. paxiana	*Ornus*	This study	BOP132410	SRR28778851		Jiuzhaigou, Sichuan, China

F. quadrangulata	*Dipetalae*	This study	ENC850132	SRR28802824	Kankakeee county,lllinois, USA
F. angustifolia	*Fraxinus*	This study	BOP132265	SRR28777866	Krym, Russia
F. longicuspis	*Ornus*	This study	ENC850147	SRR28802818	Luanchuan, Henan, China
F. malacophylla	*Ornus*	This study	POC544295	SRR28787791	Mengla, Yunnan, China
F. spaethiana	*Melioides*	This study	ENC850141	SRR28802819	Nagano-ken, Japan
F. dipetala	*Dipetalae*	This study	BOP133232	SRR28781562	Napa county, California, USA
F. anomala	*Dipetalae*	This study	BOP132267	SRR28777865	New Mexico, USA
F. velutina	*Melioides*	This study	BOP132488	SRR28781564	New Mexico, USA
F. ornus	*Ornus*	This study	BOP132402	SRR28778852	Novi Vinodolski, Croatia
F. nigra	*Fraxinus*	This study	BOP133286	SRR28802827	Ogle county, Italy
F. mandshurica	*Fraxinus*	This study	POC505349	SRR28787796	Qiangyuan, Liaoning, China
F. malacophylla	*Ornus*	This study	BOP215893	SRR28802826	Qiubei,Yunnan, China
F. sikkimensis	*Ornus*	This study	BOP132465	SRR28781566	Shangri-La, Yunnan, China
F. odontocalyx	*Ornus*	This study	BOP132400	SRR28778853	Shucheng, Anhui, China
F. griffithii	*Ornus*	This study	BOP132320	SRR28778063	Taiwan, China
F. trifoliolata	*Ornus*	This study	BOP132487	SRR28781565	Tibetan Autonomous County of Muli, Sichuan, China
F. americana	*Melioides*	This study	BOP132260	SRR18497232	Tolland county, Connecticut, USA
F. sogdiana	*Fraxinus*	This study	ENC850128	SRR28802825	Tulufan, Xinjiang, China
F. excelsior	*Fraxinus*	This study	BOP132304	SRR28778063	Valle d 'Aosta, Italian
F. bungeana	*Ornus*	This study	BOP132280	SRR28778065	Xiuwu, Henan, China
F. griffithii	*Ornus*	This study	ENC850136	SRR28802823	Yongfu, Guangxi, China
F. retusifoliolata	*Ornus*	This study	ENC850138	SRR28802821	Yunnan, China
F. angustifolia subsp. *angustifolia*	*Fraxinus*	Assembled using data from SRA		ERR3992981	
F. dipetala	*Dipetalae*	Assembled using data from SRA		ERR4007659	

F. latifolia	*Melioides*	Assembled using data from SRA	ERR4007732	
F. mandshurica	*Fraxinus*	Assembled using data from SRA	ERR4009505	
F. ornus	*Ornus*	Assembled using data from SRA	ERR4009542	
F. paxiana	*Ornus*	Assembled using data from SRA	ERR4009573	
F. pennsylvanica	*Melioides*	Assembled using data from SRA	ERR4009597	
F. pennsylvanica	*Melioides*	Assembled using data from SRA	ERR4009653	
F. quadrangulata	*Dipetalae*	Assembled using data from SRA	ERR4009705	
F. sieboldiana	*Ornus*	Assembled using data from SRA	ERR4009734	
F. velutina	*Melioides*	Assembled using data from SRA	ERR4009754	
F. angustifolia subsp. *oxycarpa*	*Fraxinus*	Assembled using data from SRA	ERR4009829	
F. angustifolia subsp. *syriaca*	*Fraxinus*	Assembled using data from SRA	ERR4009863	
F. gooddingii	*Pauciflorae*	Assembled using data from SRA	ERR4009897	
F. greggii	*Pauciflorae*	Assembled using data from SRA	ERR4009934	
F. griffithii	*Ornus*	Assembled using data from SRA	ERR4009965	
F. nigra	*Fraxinus*	Assembled using data from SRA	ERR4009998	
F. xanthoxyloides	*Sciadanthus*	Assembled using data from SRA	ERR4010168	
F. albicans	*Melioides*	Assembled using data from SRA	ERR4010315	
F. baroniana	*Ornus*	Assembled using data from SRA	ERR4010339	
F. cuspidata	*Melioides*	Assembled using data from SRA	ERR4010906	
F. chiisanensis	*Melioides*	GenBank		MF980720
F. chiisanensis	*Melioides*	GenBank		MG594385
F. lanuginosa	*Ornus*	GenBank		MH817908
F. xanthoxyloides	*Sciadanthus*	GenBank		MH817912
F. chinensis	*Ornus*	GenBank		MK299391

Supplementary Table 4. The outgroup species from GenBank used in the phylogeny analysis.

No.	Species	Accession number of GenBank
1	Forestiera philyreoides	SRR8247303
2	Ligustrum gracile	SRR8247349
3	Syringa vulgaris	SRR8247317

Supplementary Table 5. Characteristics of *Fraxinus* chloroplast genomes.

Number of accession in DNA bank/GenBank	species	%GC	Sequence Length	LSC Length	IR Length	SSC Length
ERR4010315	F. albicans	37.9	155600	86429	25703	17765
BOP132260	F. americana	37.9	155601	86430	25703	17765
BOP132265	F. angustifolia	37.8	155803	86576	25705	17817
ERR3992981	F. angustifolia subsp. angustifolia	37.8	155607	86342	25705	17855
ERR4009829	F. angustifolia subsp. oxycarpa	37.8	155590	86356	25705	17824
ERR4009863	F. angustifolia subsp. syriaca	37.8	155564	86326	25711	17816
BOP132267	F. anomala	37.9	155595	86429	25696	17774
ERR4010339	F. baroniana	37.9	155686	86436	25703	17844
POC516242	F. baroniana	37.9	155691	86441	25703	17844
BOP132272	F. berlandierana	37.9	155629	86486	25685	17773
POC516247	F. bungeana	37.9	155639	86417	25709	17804
BOP132280	F. bungeana	37.9	155634	86412	25709	17804
MF980720	F. chiisanensis	37.9	155572	86411	25697	17767
MG594385	F. chiisanensis	37.9	155542	86385	25695	17767
POC506018	F. chinensis	37.9	155639	86417	25709	17804
MK299391	F. chinensis	37.9	155639	86417	25709	17804
BOP132451	F. chinensis subsp. rhynchophylla	37.9	155766	86537	25711	17806
ERR4010906	F. cuspidata	37.8	155463	86283	25703	17774
BOP133232	F. dipetala	37.9	155616	86429	25698	17791
ERR4007659	F. dipetala	37.9	155610	86424	25698	17790
BOP132348	F. dipetala	37.9	155568	86384	25698	17788
BOP132304	F. excelsior	37.8	155573	86333	25704	17832
POC516253	F. excelsior	37.8	155573	86333	25704	17832
ERR4009897	F. gooddingii	37.9	155629	86452	25700	17777
ERR4009934	F. greggii	37.9	155563	86403	25701	17758
BOP132320	F. griffithii	37.9	155690	86473	25693	17831
ERR4009965	F. griffithii	37.9	155682	86465	25693	17831
BOP132327	F. griffithii	37.9	155674	86442	25703	17826
ENC850136	F. griffithii	37.9	155651	86431	25697	17826
POC516254	F. hupehensis	37.9	155650	86463	25692	17803
BOP132443	F. insularis	37.9	155704	86472	25703	17826
ENC850137	F. insularis	37.9	155705	86473	25704	17824
BOP132360	F. lanuginosa	37.9	155630	86414	25706	17804
MH817908	F. lanuginosa	37.9	155628	86412	25706	17804
ERR4007732	F. latifolia	37.8	155611	86485	25683	17760

ENC850147	F. longicuspis	37.9	155686	86453	25703	17827
BOP215893	F. malacophylla	37.9	155620	86404	25697	17822
POC544295	F. malacophylla	37.9	155612	86396	25697	17822
ERR4009505	F. mandshurica	37.8	155558	86330	25705	17818
POC505349	F. mandshurica	37.8	155590	86362	25705	17818
BOP133286	F. nigra	37.8	155344	86116	25705	17818
ERR4009998	F. nigra	37.8	155346	86118	25705	17818
BOP132400	F. odontocalyx	37.9	155685	86451	25704	17826
BOP132402	F. ornus	37.9	155655	86447	25696	17816
ERR4009542	F. ornus	37.8	155682	86473	25698	17813
BOP132410	F. paxiana	37.9	155203	85986	25706	17805
ERR4009573	F. paxiana	37.9	155207	85990	25706	17805
POC516251	F. pennsylvanica	37.8	155611	86485	25683	17760
ERR4009597	F. pennsylvanica	37.8	155601	86475	25683	17760
ERR4009653	F. pennsylvanica	37.8	155592	86466	25682	17762
BOP132432	F. platypoda	37.9	155532	86377	25694	17767
ENC850132	F. quadrangulata	37.8	155541	86403	25681	17776
ERR4009705	F. quadrangulata	37.8	155541	86403	25681	17776
ENC850138	F. retusifoliolata	37.9	155615	86386	25703	17823
ERR4009734	F. sieboldiana	37.8	155697	86465	25704	17824
BOP132465	F. sikkimensis	37.9	155687	86476	25704	17803
ENC850128	F. sogdiana	37.8	155597	86388	25697	17815
ENC850141	F. spaethiana	37.9	155615	86407	25713	17782
ENC850139	F. texensis	37.9	155667	86495	25704	17764
BOP132487	F. trifoliolata	37.9	155699	86472	25704	17819
ERR4009754	F. velutina	37.9	155453	86314	25687	17765
BOP132488	F. velutina	37.8	155608	86477	25683	17765
BOP132490	F. velutina var. coriacea	37.9	155580	86447	25685	17763
ERR4010168	F. xanthoxyloides	37.9	155689	86500	25692	17805
MH817912	F. xanthoxyloides	37.9	155694	86507	25692	17803

Supplementary Table 6. Fossil records and priors' parameters for divergence estimate.

Type	Age	Tip-dating ages	Locality	Taxa	Section	Prior distribution	Parameters	Reference
Calibration point	Early Eocene	52.5 Ma	-	-	-	Normal	Mean=0.0 Sigma=1.0 Offset=52.5	Dong et al. 2022
Fossil	Early Eocene	51.5 Ma	Quilchena locality, British Columbia, Canada	*Fraxinus eoemarginata* Mathewes, S.B. Archibald et A. Lundgren	not certain	Lognormal	M=1.0 S=0.5 Offset=51.5	Mathewes et al. 2021
Fossil	Early Oligocene	33.0 Ma	Lühe, Yunnan, China	*Fraxinus zlatkoi* Meng-Xiao Wu et J. Huang	*Dipetalae*	Lognormal	M=1.0 S=0.5 Offset=33.0	Wu et al. 2022
Fossil	Early Oligocene	33.0 Ma	Lühe, Yunnan, China	*Fraxinus* cf. *honshuensis* Tanai et Onoe	*Ornus*	Lognormal	M=1.0 S=0.5 Offset=33.0	Wu et al. 2022
Fossil	Oligocene	31.4 Ma	Longjing, Jilin, China	*Fraxinus honshuensis* Tanai et Onoe	*Ornus*	Lognormal	M=1.0 S=0.5 Offset=31.4	Axelrod 1998
Fossil	Middle Miocene	16.3 Ma	Shanwang, Shandong, China	*Fraxinus dayana* R.W.Chaney et Axelrod	*Melioides*	Lognormal	M=1.0 S=0.5 Offset=16.3	Yang and Yang 1994

Fossil	Late Pliocene	2.1 Ma	Bernasso, France	*Fraxinus ornus* L.	*Ornus*	Lognormal	M=1.0<br>S=0.5<br>Offset=2.1	Leroy and Roiron 1996

Supplementary Table 7. Samara traits from extant species and fossil records.

Fossil/Species	Group	Fruit Length	Fruit Width	Seed Length	Seed Width	FL/FW	SL/SW	FL/SL
F. zlatkoi	Dipetalae	18.9	6	8.5	3.8	3.15	2.24	2.22
F. cf. honshuensis	Ornus	20.85	4.1	7.4	2.1	5.09	3.52	2.82
F. dayana	Melioides	52.5	10	14.5	3.5	5.25	4.14	3.62
F. honshuensis	Ornus	20	4	8	2	5.00	4.00	2.50
F. anomala	Dipetalae	20.9	6.8	13.1	4.35	3.07	3.01	1.60
F. dipetala	Dipetalae	24.05	6.05	13.95	4.6	3.98	3.03	1.72
F. americana	Melioides	35.35	4.55	11.85	2.8	7.77	4.23	2.98
F. platypoda	Melioides	57.25	9.8	26.55	6.75	5.84	3.93	2.16
F. purpusii	Pauciflorae	21.8	5.2	9.45	3.2	4.19	2.95	2.31
F. hubeiensis	Sciadanthus	42.2	8.9	15.3	4.05	4.74	3.78	2.76
F. xanthoxyloides	Sciadanthus	29.8	5.75	14.25	1.95	5.18	7.31	2.09
F. excelsior	Fraxinus	28.75	7.2	16.9	5.15	3.99	3.28	1.70
F. mandshurica	Fraxinus	32.45	7.65	16.05	5.55	4.24	2.89	2.02
F. chinensis	Ornus	32.8	5.9	12.6	2.15	5.56	5.86	2.60
F. malacophylla	Oruns	23.3	4.3	8.75	1.8	5.42	4.86	2.66

Supplementary Table 8. Results of model tests. Bolded are the final models used in the analysis.

Phylogeny	Models	LnL	Numparams	AICc	AICc_wt
Tip-dated	**DEC**	**-127.7**	**2**	**259.6**	**1**
	DIVALIKE	-138.3	2	280.9	2.40E-05
	BAYAREALIKE	-142.8	2	289.8	2.80E-07
Node-dated	DEC+J	-113.8	3	233.5	0.98
	DIVALIKE+J	-122.3	3	250.6	0.7
	**BAYAREALIKE+J**	**-108.3**	**3**	**222.6**	**1**
	DEC	-118.6	2	241.2	0.021
	DIVALIKE	-124.1	2	252.3	0.3
	BAYAREALIKE	-121.6	2	247.1	4.70E-06

Supplementary Table 9. Dispersal constraints between different regions.

		WNA	ENA	EU	EA	CA	JP
5-0Ma	**WNA**	1	1	0.01	0.5	0.01	0.01
	**ENA**	1	1	0.25	0.01	0.01	0.01
	**EU**	0.01	0.25	1	1	1	0.01
	**EA**	0.5	0.01	1	1	1	0.5
	**CA**	0.01	0.01	1	1	1	0.01
	**JP**	0.01	0.01	0.01	0.5	0.01	1
30-5Ma	**WNA**	1	1	0.01	1	0.01	0.01
	**ENA**	1	1	0.5	0.01	0.01	0.01
	**EU**	0.01	0.5	1	1	1	0.01
	**EA**	1	0.01	1	1	1	0.75
	**CA**	0.01	0.01	1	1	1	0.01
	**JP**	0.01	0.01	0.01	0.75	0.01	1
52-30Ma	**WNA**	1	1	0.01	1	0.01	0.01
	**ENA**	1	1	1	0.01	0.01	0.01
	**EU**	0.01	1	1	0.01	0.01	0.01
	**EA**	1	0.01	0.01	1	1	0.75
	**CA**	0.01	0.01	0.01	1	1	0.01
	**JP**	0.01	0.01	0.01	0.75	0.01	1

Supplementary Table 10. Raw data for the OLS analysis.

Time	Parameters	ABSR2	Dispersal/Vicariance	Intercontinental Event
1.061	1.261	0.761	Vicariance	FASLE
4.474	2.543	1.135	Vicariance	TRUE
3.135	2.498	1.242	Vicariance	FASLE
3.905	2.672	0.897	Vicariance	TRUE
6.603	1.229	0.264	Vicariance	TRUE
21.733	0.929	0.461	Vicariance	TRUE
34.457	1.088	0.969	Vicariance	FASLE
39.586	0.997	0.397	Vicariance	TRUE
32.757	0.377	0.681	Vicariance	FASLE
53.965	1.144	0.191	Vicariance	FASLE
0.357	7.044	0.777	Dispersal	FASLE
0.529	7.100	0.737	Dispersal	FASLE
0.895	7.122	0.748	Dispersal	FASLE
0.895	7.122	0.748	Dispersal	FASLE
0.927	7.117	0.654	Dispersal	FASLE
1.897	6.651	0.786	Dispersal	FASLE
2.076	6.450	0.720	Dispersal	FASLE
4.032	5.912	1.225	Dispersal	FASLE
4.345	6.219	1.207	Dispersal	FASLE
4.925	7.031	1.463	Dispersal	FASLE
5.274	7.282	1.406	Dispersal	FASLE
5.815	7.482	1.184	Dispersal	FASLE
6.094	7.488	1.208	Dispersal	FASLE
6.603	7.319	1.264	Dispersal	FASLE
7.560	6.802	0.550	Dispersal	FASLE
7.902	6.450	0.518	Dispersal	FASLE
8.558	5.505	0.363	Dispersal	FASLE
8.737	5.121	0.352	Dispersal	FASLE
8.864	4.825	0.147	Dispersal	FASLE
13.193	0.916	0.683	Dispersal	FASLE
16.557	0.932	0.710	Dispersal	FASLE
22.353	1.075	0.709	Dispersal	FASLE
24.709	1.078	0.420	Dispersal	FASLE
32.757	4.339	0.681	Dispersal	TRUE
34.188	6.370	1.275	Dispersal	TRUE
34.457	6.247	1.169	Dispersal	TRUE
34.695	5.985	1.254	Dispersal	TRUE
51.400	0.931	0.181	Dispersal	TRUE

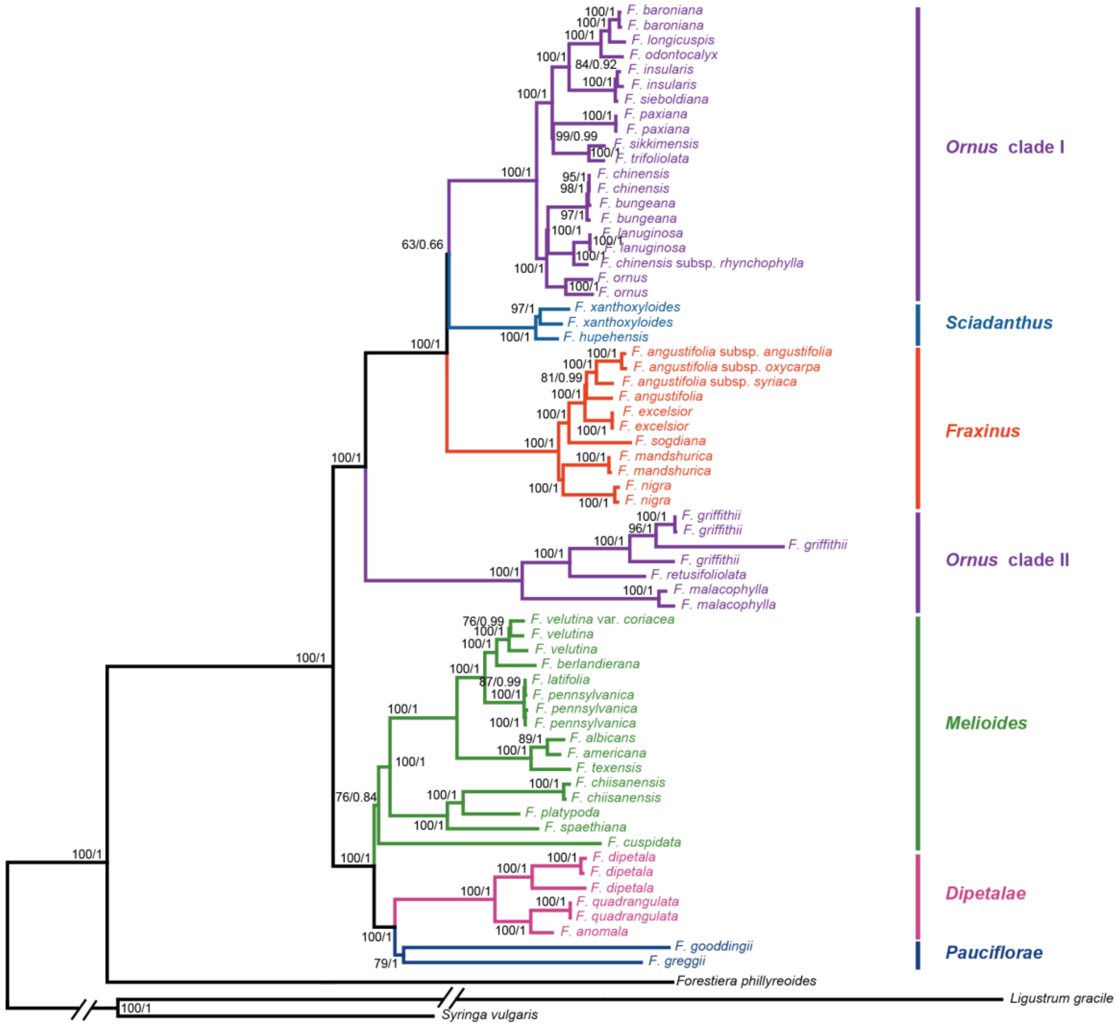

Supplementary Figure 1. Phylogenetic tree with branch lengths of *Fraxinus* inferred using the chloroplast genome dataset in Raxml-NG. Numbers associated with nodes indicate ML bootstrap support (BS) values and BI posterior probabilities (PP). The different colours represent the six sections of *Fraxinus* according to the traditional classification.

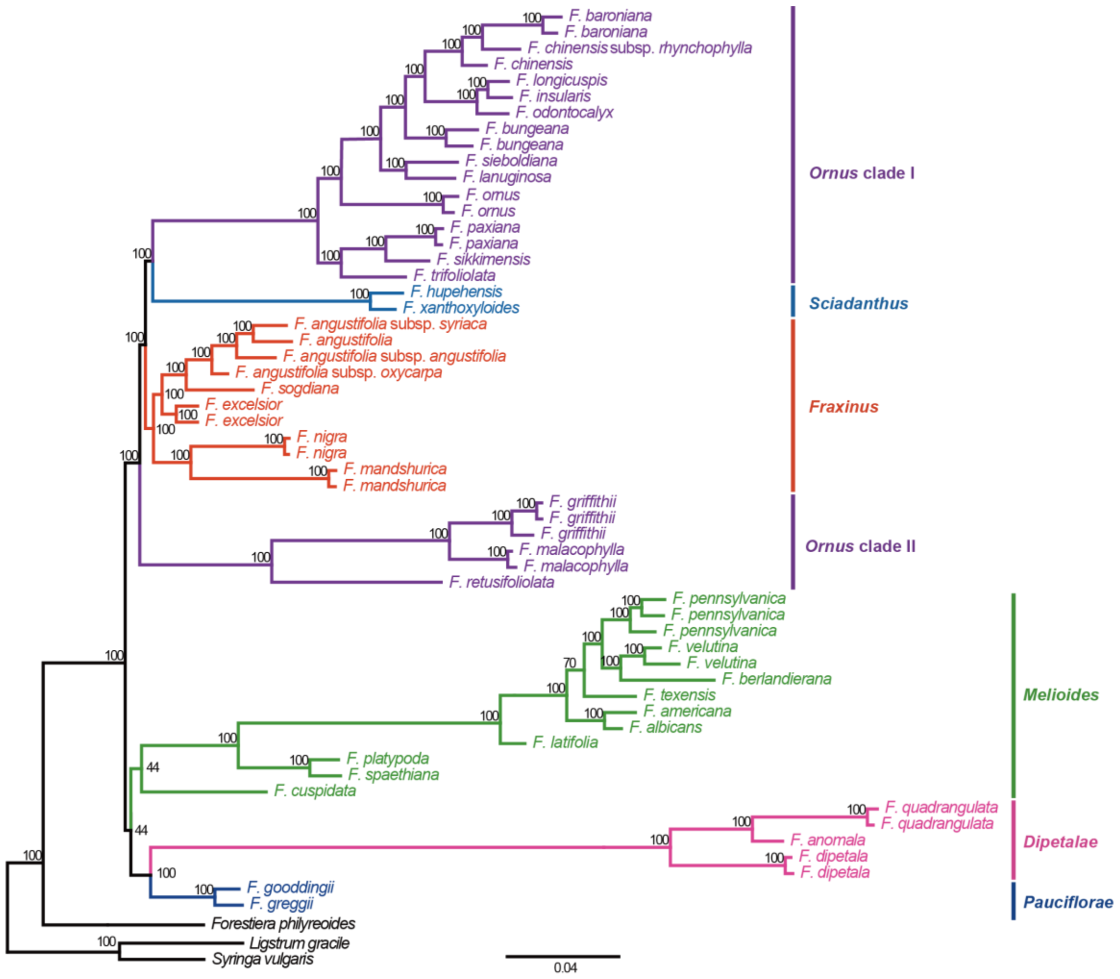

Supplementary Figure 2. Phylogenetic tree with branch lengths of *Fraxinus* inferred using the SNP dataset in Raxml-NG. Numbers associated with nodes indicate ML bootstrap support (BS) values. The different colours represent the five six sections of Fraxinus according to the traditional classification.

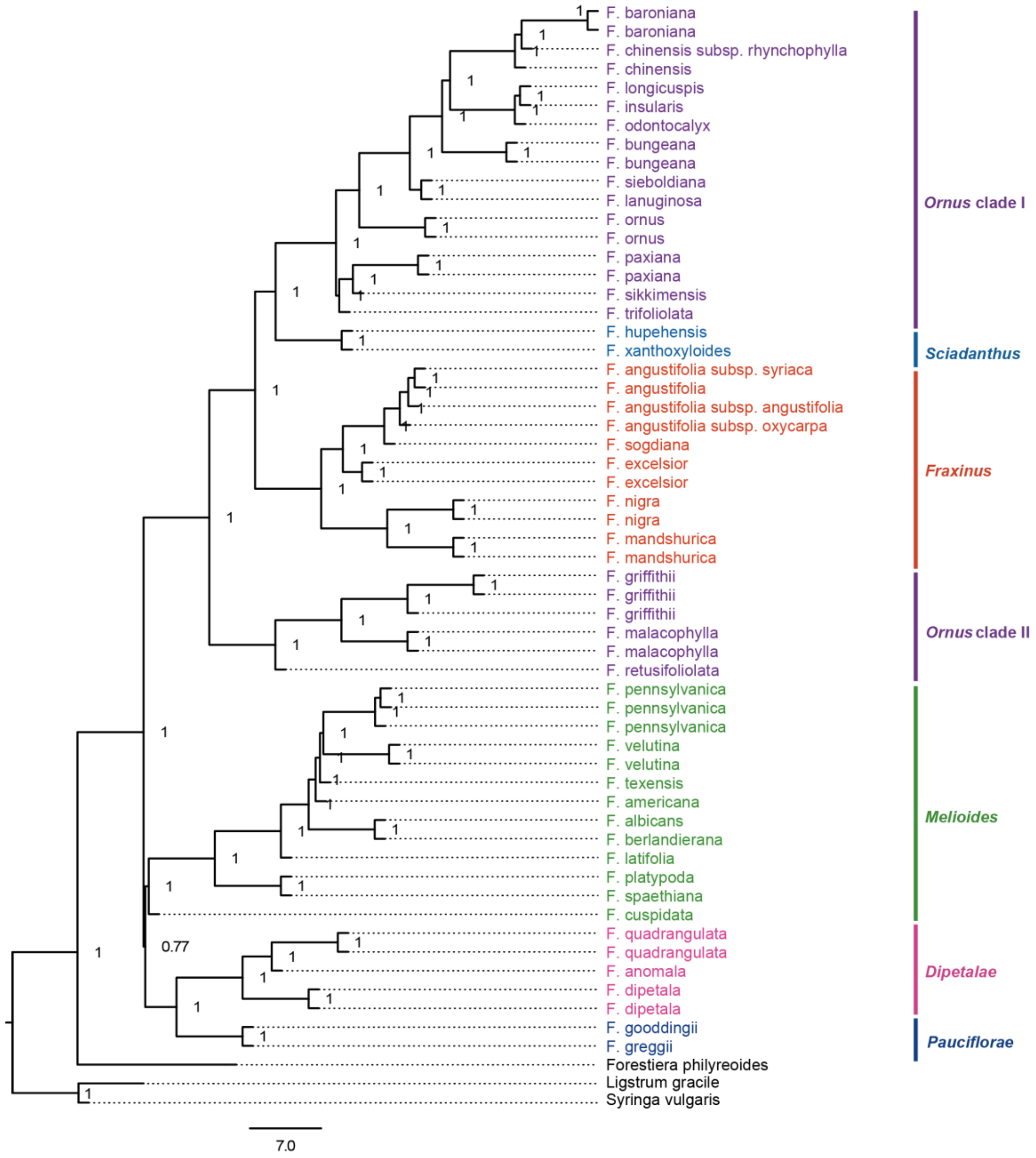

Supplementary Figure 3. Species tree based on the 5kb SNPs dataset.

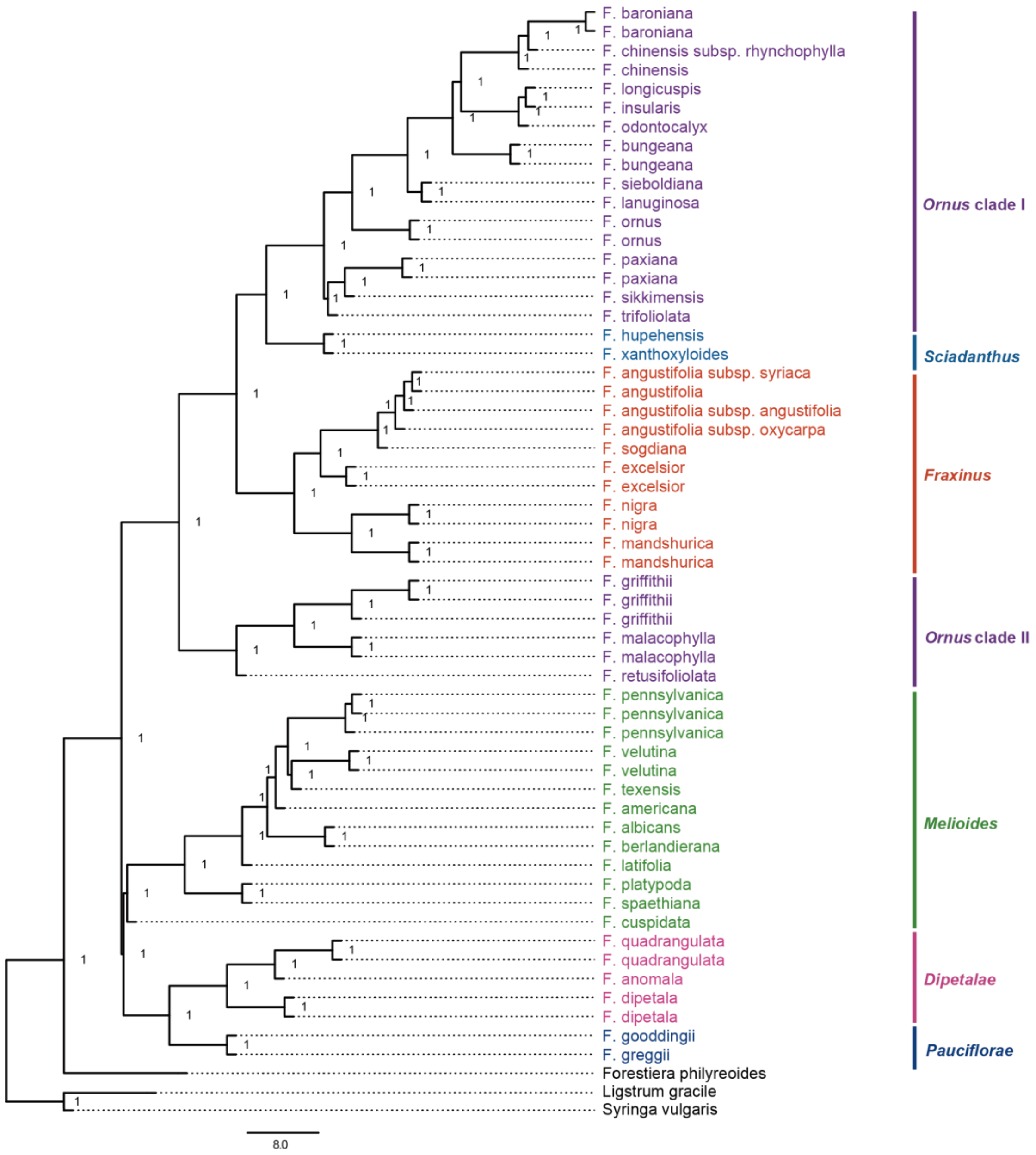

Supplementary Figure 4. Species tree based on the 10kb SNPs dataset.

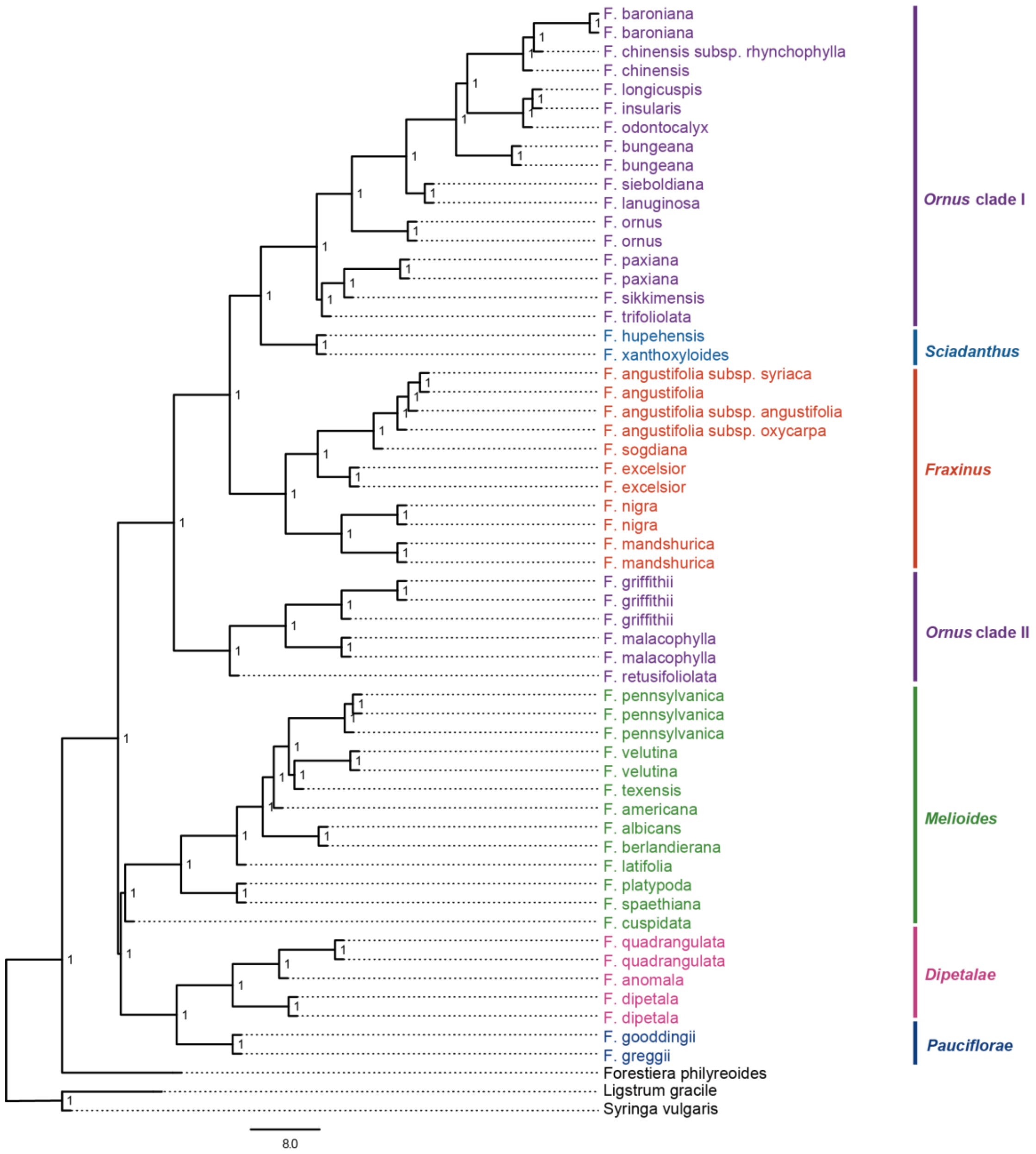

Supplementary Figure 5. Species tree based on the 15kb SNPs dataset.

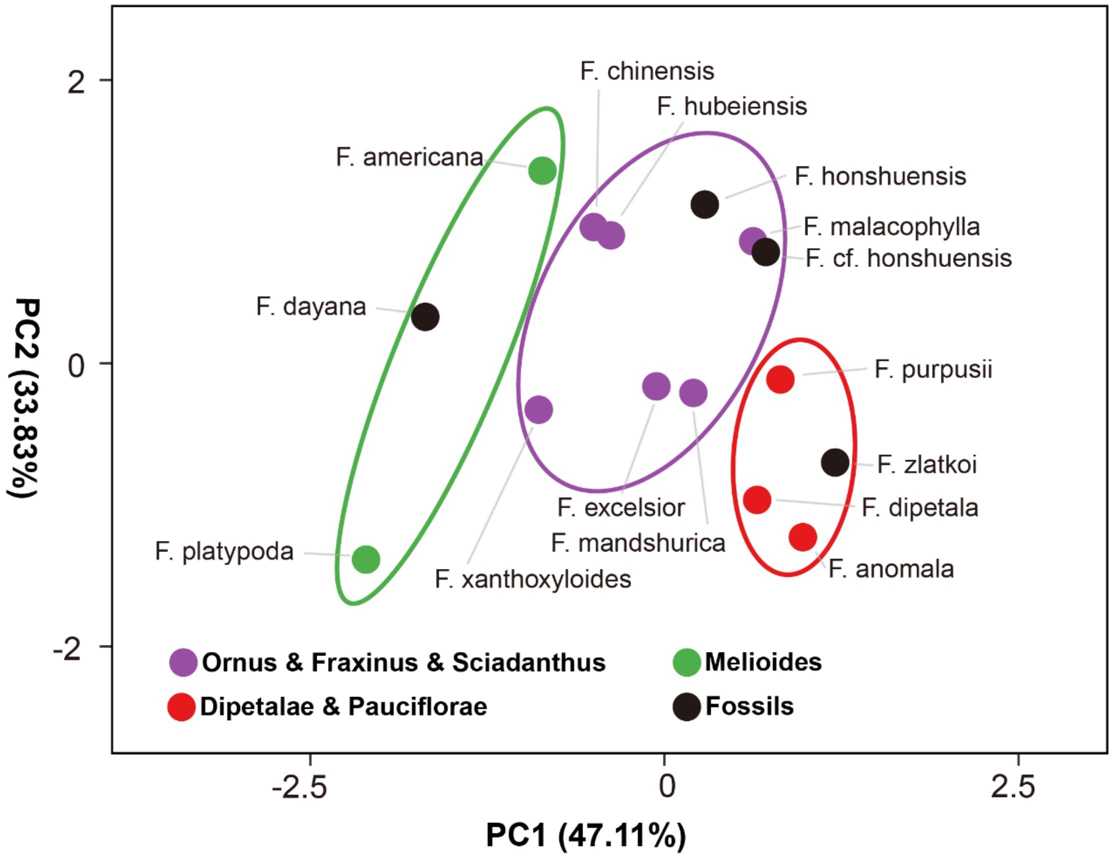

Supplementary Figure 6. PCA results for fossils' location in phylogeny. Different colors represent representative species corresponding to different clades in phylogeny. Black dots refer to the fossil species used in our research.

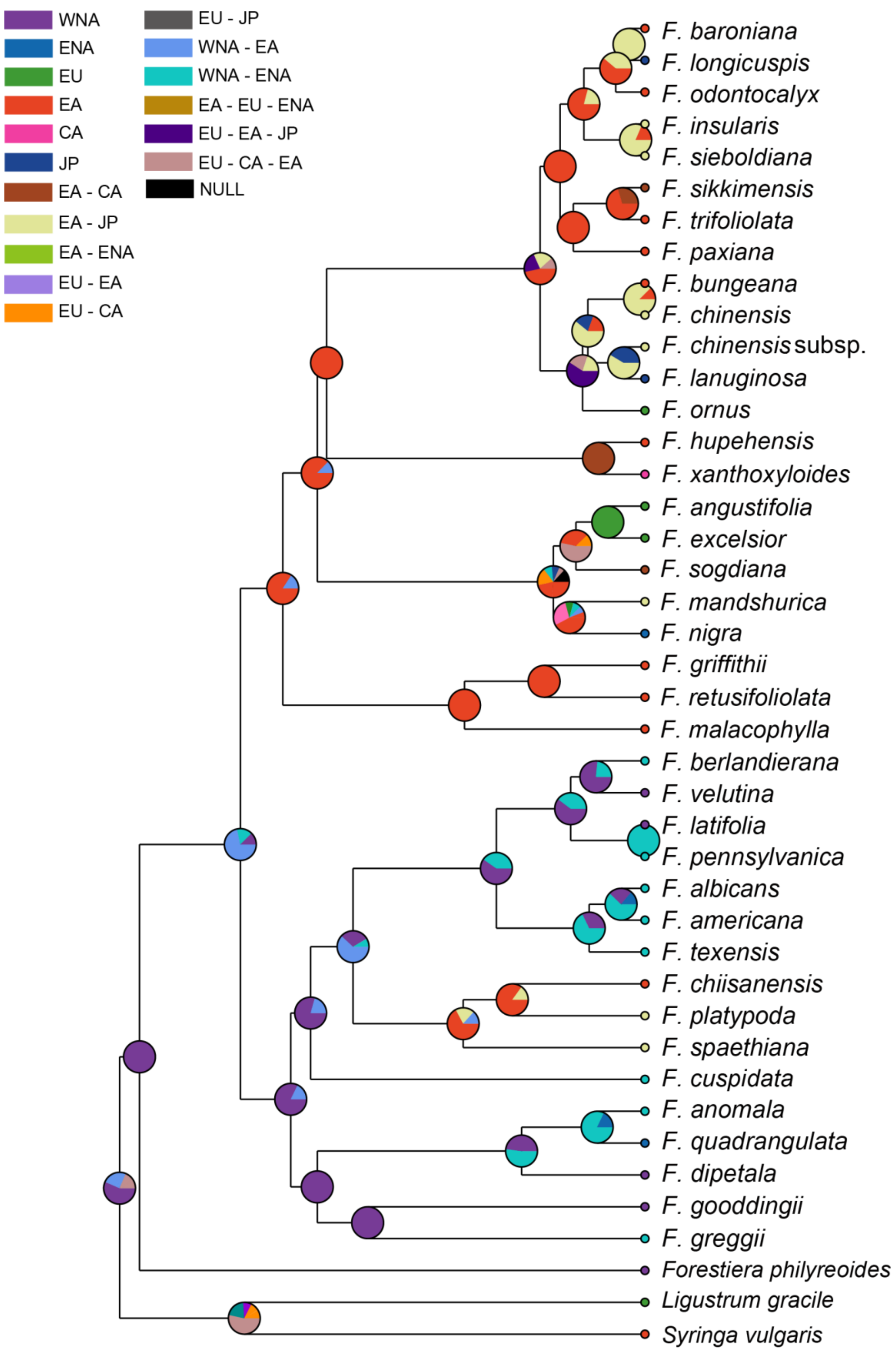

Supplementary Figure 7. Ancestral area reconstruction based on node-dating phylogeny with DEC model.

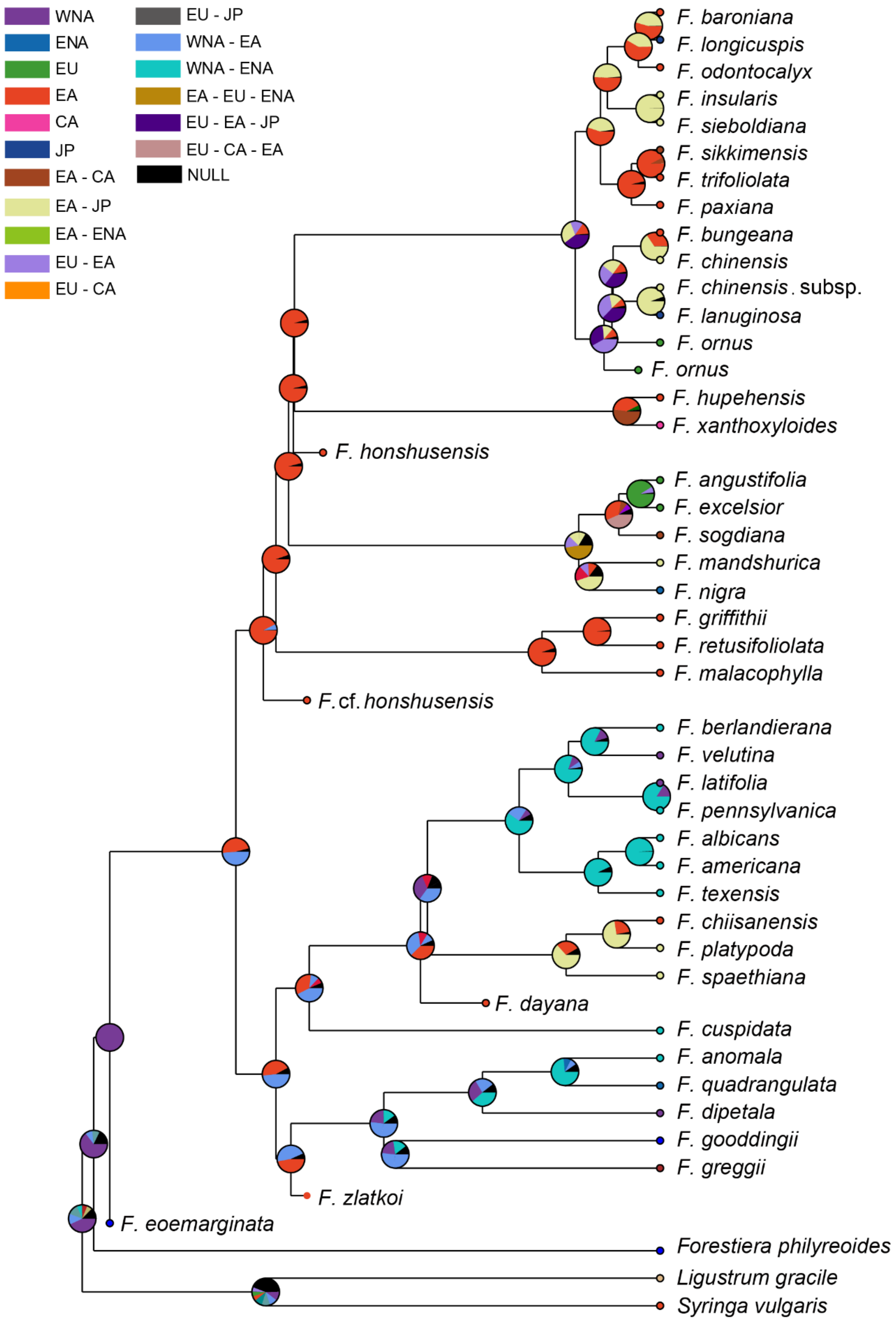

Supplementary Figure 8. Ancestral area reconstruction based on tip-dating phylogeny with BAYAREALIKE+J model.

Supplementary References

Axelrod D I. The Oligocene Haynes creek flora of eastern Idaho[M]. Univ of California Press, 1998.

Becker H F. Fossil plants of the Tertiary Beaverhead Basins in southwestern Montana[J]. Palaeontographica Abteilung B, 1969: 1-142.

Bruch A A, Zhilin S G. Early Miocene climate of Central Eurasia—evidence from Aquitanian floras of Kazakhstan[J]. Palaeogeography, Palaeoclimatology, Palaeoecology, 2007, 248(1-2): 32-48.

Call V B, Dilcher D L. Investigations of angiosperms from the Eocene of southeastern North America: samaras of Fraxinus wilcoxiana Berry[J]. Review of Palaeobotany and Palynology, 1992, 74(3-4): 249-266.

Grímsson F, Denk T, Símonarson L A. Middle Miocene floras of Iceland—the early colonization of an island?[J]. Review of Palaeobotany and Palynology, 2007, 144(3-4): 181-219.

Jung S H, Lee S J. Fossil-Winged Fruits of Fraxinus (Oleaceae) and Liriodendron (Magnoliaceae) from the Duho Formation, Pohang Basin, Korea[J]. Acta Geologica Sinica-English Edition, 2009, 83(5): 845-852.

Kvaček Z, Manchester S R, Guo S. Trifoliolate leaves of Platanus bella (Heer) comb. n. from the Paleocene of North America, Greenland, and Asia and their relationships among extinct and extant Platanaceae[J]. International Journal of Plant Sciences, 2001, 162(2): 441-458.

Kvaček Z, Teodoridis V. Tertiary macrofloras of the Bohemian Massif: a review with correlations within Boreal and Central Europe[J]. Bulletin of Geosciences, 2007, 82(4): 383-408.

Kvaček Z, Walther H. The Oligocene volcanic flora of Kundratice near Litoměřice, České Středohoří volcanic complex (Czech Republic): a review[M]. Národní muzeum, 1998.

Leroy S A G, Roiron P. Latest Pliocene pollen and leaf floras from Bernasso palaeolake (Escandorgue Massif, Hérault, France)[J]. Review of Palaeobotany and Palynology, 1996, 94(3-4): 295-328.

Mathewes R, Archibald S B, Lundgren A. Tips and identification of early Eocene Fraxinus L. samaras from the Quilchena locality, Okanagan Highlands, British Columbia, Canada[J]. Review of Palaeobotany and Palynology, 2021, 293: 104480.

Meyer H W. The Oligocene bridge creek flora of the john day formation, Oregon[J]. University of California Publications in Geological Science, 1997, 141: 1.

Tanai T. Late Tertiary floras from northeastern Hokkaido, Japan[J]. Palaeont. Soc. Japan, Spec. Pap., 1965, 10: 1-117.

Yang H, Yang S. The Shanwang fossil biota in eastern China: a Miocene Konservat-Lagerstätte in lacustrine deposits[J]. Lethaia, 1994, 27(4): 345-354.

Zhilin S G. History of the development of the temperate forest flora in Kazakhstan, USSR from the Oligocene to the early Miocene[J]. The Botanical Review, 1989, 55: 205-330.
